# Intelligent segmentation of thyroid nodule ultrasound images based on benign–malignant-aware semantic prototype calibration and class-conditional boundary refinement

**DOI:** 10.3389/fcell.2026.1879094

**Published:** 2026-08-21

**Authors:** Bin Luo, Runwen Li, Yao Tang

**Affiliations:** 1 Department of General Surgery, Panzhihua Central Hospital, Panzhihua, Sichuan, China; 2 Department of Vascular Diseases, Panzhihua Central Hospital, Panzhihua, Sichuan, China; 3 Department of Ophthalmology, Panzhihua Central Hospital, Panzhihua, Sichuan, China

**Keywords:** benign–malignant-aware segmentation, boundary refinement, semantic prototype calibration, thyroid nodule, ultrasound image segmentation

## Abstract

**Introduction:**

Ultrasound-based thyroid nodule segmentation remains challenging because of blurred boundaries, complex echo noise, and subtle morphological differences between benign and malignant lesions.

**Methods:**

We propose a benign-malignant-aware segmentation framework based on SegFormer, incorporating adaptive semantic prototype calibration (ASPC) for class-level semantic discrimination and class-conditional boundary refinement (CCBR) for ambiguous contour correction. The model performs three-class segmentation of background, benign, and malignant regions.

**Results:**

Experiments on the public TN3K dataset and an in-house clinical dataset demonstrate consistent performance under both external transfer and supervised evaluation settings. The proposed method achieved PA, mIoU, mDice, and mPrecision of 0.9449, 0.6427, 0.7517, and 0.8147 on TN3K; 0.7793, 0.5016, 0.6374, and 0.7089 under direct external transfer; and 0.8521, 0.6124, 0.7328, and 0.7996 under supervised in-house evaluation.

**Discussion:**

Ablation studies and Grad-CAM visualization further confirm that ASPC and CCBR improve semantic discrimination, boundary refinement, and model interpretability, supporting the effectiveness of the proposed framework for thyroid nodule segmentation.

## 1 Introduction

Thyroid nodules are common lesions in clinical ultrasound examinations. Accurate identification of nodule location, boundary extent, and benign or malignant tendency is important for risk assessment, follow-up management, and treatment planning. Ultrasound imaging is widely used for thyroid nodule screening because it is non-invasive, real-time, and low-cost. However, thyroid ultrasound images often have low contrast, speckle noise, blurred boundaries, and complex tissue echoes. These factors make manual delineation subjective and difficult. In recent years, deep learning methods have been widely used for segmentation of thyroid nodule ultrasound images. Sun et al. (2022) proposed TNSNet with soft shape supervision to enhance shape constraints for nodule regions. Li C. et al. (2023) designed a novel model for thyroid nodule ultrasound image segmentation to improve nodule localization performance. Bi et al. (2023) proposed BPAT-UNet, which improves boundary modeling through a boundary-preserving mechanism and a Transformer architecture. Gong et al. (2023) introduced a thyroid-region-prior-guided attention mechanism to strengthen the model’s focus on nodule-related regions. In addition, Chen et al. (2024) proposed the multi-view learning segmentation model MLMSeg, and Yang et al. (2024) constructed a lightweight attention segmentation network DAC-Net. These studies have promoted the development of automatic segmentation for thyroid nodule ultrasound images.

Although existing methods have made progress in nodule region segmentation, several limitations remain. First, most methods treat thyroid nodules as a single foreground target. They mainly focus on the boundary between the nodule and the surrounding background tissues. Benign–malignant information is rarely modeled in a unified segmentation label space. Kang et al. (2022) explored consistency learning between thyroid nodule segmentation and classification, and Li G. et al. (2023) fused Transformer and large-kernel convolution for malignant thyroid nodule segmentation. However, benign–malignant semantic differences are still not fully incorporated into most segmentation frameworks. Second, benign and malignant nodules often show fine-grained differences in ultrasound images. These differences may appear in edge regularity, internal echoes, local heterogeneous textures, and boundary transition patterns. It is difficult to represent these subtle differences using ordinary foreground segmentation alone. Third, although existing methods have introduced context modeling, frequency-domain enhancement, class representation attention, and densely connected feature learning (Chen et al., 2023; Sun et al., 2024; Ali et al., 2024; Ozcan et al., 2024), they may still produce under-segmentation, over-segmentation, or unstable category responses in boundary-uncertain and benign–malignant confusing regions. Therefore, it remains necessary to improve nodule localization, benign–malignant semantic discrimination, and boundary correction in a unified framework.

To address the above problems, this article proposes a benign–malignant-aware segmentation framework for thyroid ultrasound images. The framework adopts SegFormer as the backbone network. Its hierarchical encoding structure is used to extract multi-scale semantic features. The original binary nodule annotations and image-level benign–malignant labels are jointly modeled to construct a three-class class-conditional segmentation task, including background, benign nodules, and malignant nodules. An adaptive semantic prototype calibration module is designed on this basis. It learns category-level semantic prototypes for the background, benign nodules, and malignant nodules. It also strengthens benign–malignant discrimination by matching pixel features and category prototypes. Furthermore, a class-conditional boundary refinement module is proposed. This module fuses basic prediction responses, prototype-calibrated responses, predictive entropy uncertainty, and foreground boundary responses. It then performs dynamic residual correction for low-contrast boundaries, weak-texture regions, and category-confusing regions. Using these modules, the model can localize thyroid nodule regions and provide class-conditional segmentation results for benign and malignant nodule categories.

The main contributions of this article are summarized as follows:

- A benign–malignant-aware thyroid ultrasound image segmentation framework is constructed under a class-conditional segmentation setting. The framework uses the dataset-provided nodule masks and image-level benign–malignant labels to jointly model background, benign nodules, and malignant nodules, so that nodule localization and category-aware region discrimination can be evaluated in a unified segmentation space.
- A task-specific adaptive semantic prototype calibration module is designed. The module introduces category-level prototypes for background, benign nodules, and malignant nodules, and explicitly supervises the prototype-calibrated logits using the class-conditional label map. This design strengthens the correspondence between prototypes and semantic categories and improves benign and malignant region discrimination.
- A class-conditional boundary refinement module is developed for uncertain and category-confusing regions. By jointly using basic prediction logits, prototype-calibrated responses, predictive entropy, and foreground boundary responses, the module performs adaptive residual correction and improves boundary consistency in thyroid ultrasound segmentation.

## 2 Datasets

### 2.1 TN3K dataset

**FIGURE 1:**
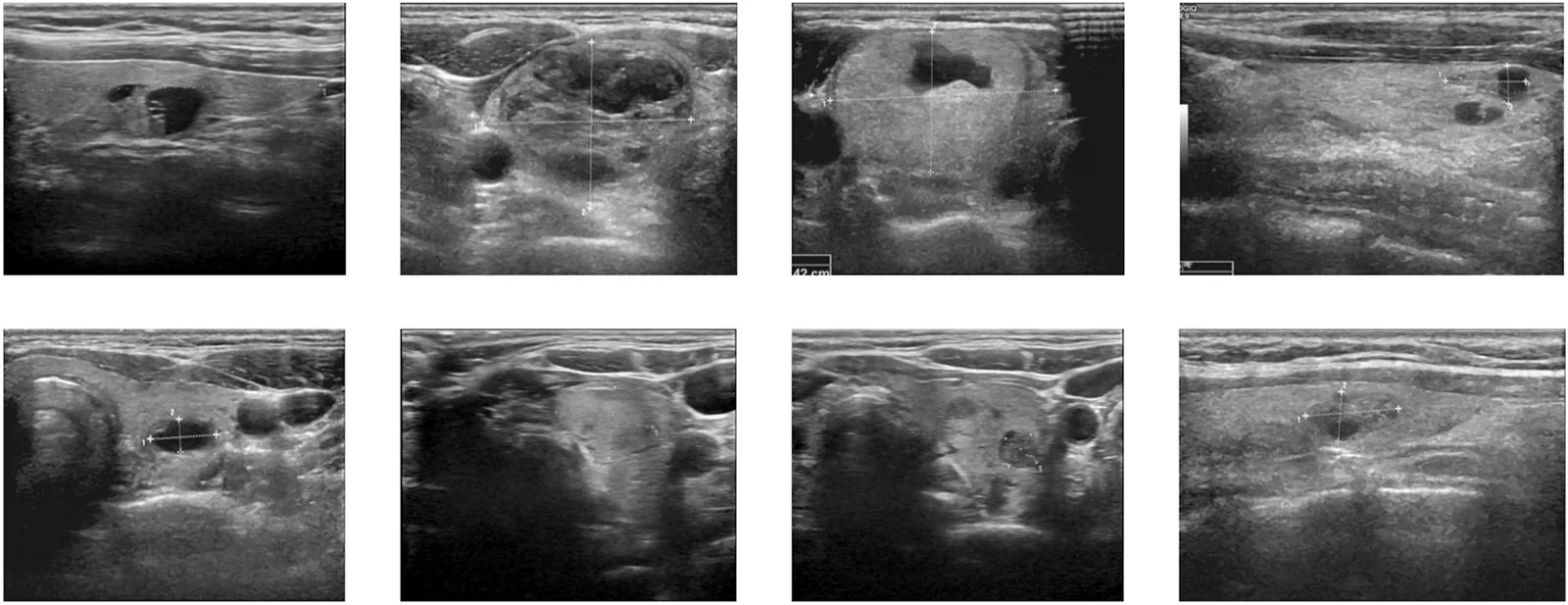
TN3K dataset examples.

The experiments in this study were conducted on the publicly available TN3K thyroid ultrasound image dataset for model training and testing. This dataset contains thyroid nodule ultrasound images, pixel-level binary nodule segmentation annotations, and image-level benign–malignant labels provided by the original dataset, thereby supporting thyroid nodule localization and class-conditional benign–malignant nodule segmentation rather than true intra-tumoral pixel-level malignancy distribution modeling. To ensure consistency with the experimental setting of the open-source dataset, this study strictly follows the official split provided by the TN3K dataset for the training-validation set and test set. Specifically, the training and validation samples are obtained from trainval-image and trainval-mask. The test samples are obtained from test-image and test-mask, without additionally re-splitting the test set. In the data preprocessing stage, the original binary nodule masks are first matched with the image-level benign–malignant labels provided by the TN3K annotations. Accordingly, the background region is labeled as 0, the nodule region from benign-labeled images is labeled as 1, and the nodule region from malignant-labeled images is labeled as 2. Therefore, the constructed labels represent class-conditional nodule masks guided by image-level benign–malignant labels, rather than pixel-level pathological heterogeneity within the nodule. Because a single image-level benign–malignant label cannot be uniquely assigned to each annotated nodule in an image containing multiple nodules, such multi-nodule samples were excluded during preprocessing to reduce label ambiguity and potential label noise. Subsequently, all input images and labels are uniformly resized to 512×512 . Bilinear interpolation is used for images, and nearest-neighbor interpolation is used for masks to avoid damaging category values. Finally, image normalization is performed, and random horizontal flipping, brightness perturbation, and slight noise augmentation are used during training to improve the generalization ability of the model under different ultrasound imaging qualities and nodule morphologies. Examples from the dataset are shown in Figure 1.

### 2.2 Clinical validation dataset

To further verify the generalization ability of the model in real clinical scenarios, this study constructed a thyroid nodule ultrasound image validation dataset derived from clinical practice. This dataset was reviewed and approved by the hospital ethics committee, with the ethics approval number Panlun [REC-2024-007]. The research process followed the basic ethical principles for medical research stated in the Declaration of Helsinki and strictly implemented the requirements for subject privacy protection and data anonymization. Because this study is a retrospective medical image analysis study, all data used in this work were collected from thyroid ultrasound images acquired during previous routine clinical diagnosis and treatment. No additional interventions were performed on the clinical diagnosis and treatment process; no additional risk was introduced to the subjects; and all data had been de-identified before database construction and analysis. Therefore, the requirement for informed consent was waived after ethics review and approval. The clinical validation dataset included 73 thyroid nodule cases: 53 benign and 20 malignant. Eight representative ultrasound images were selected for each case, resulting in a total of 584 images. All images were independently annotated by two expert physicians with experience in thyroid ultrasound diagnosis. When disagreements occurred, a consensus annotation was obtained through review and discussion. To avoid data leakage caused by images from the same case appearing in different data subsets, this study strictly divided the training, validation, and test sets at the case level. That is, the eight images selected from the same case were all assigned to the same subset, and only to that subset. The case composition and split of the clinical validation dataset are shown in Table 1.

**TABLE 1:** Case composition and case-level split of the clinical validation dataset.

Data subset	Number of cases	Benign cases	Malignant cases	Number of ultrasound images
Training set	51	37	14	408
Validation set	11	8	3	88
Test set	11	8	3	88
Total	73	53	20	584

## 3 Methodology

### 3.1 Overall model architecture

**FIGURE 2:**
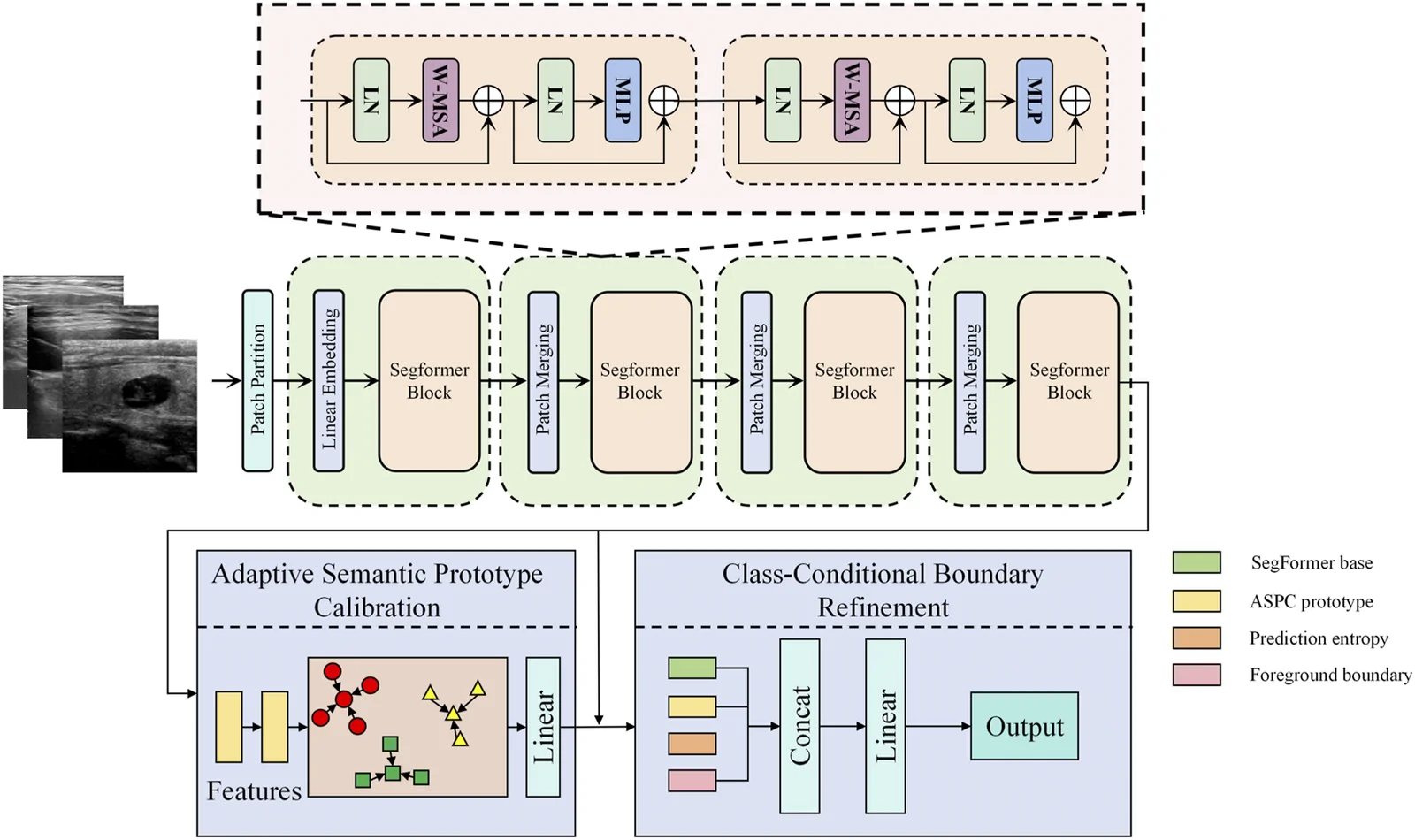
The proposed benign–malignant-aware segmentation framework for thyroid ultrasound images consists of a hierarchical SegFormer encoder, an adaptive semantic prototype calibration module, and a class-conditional boundary refinement module. The model enhances the categorical discriminability of benign and malignant nodule regions through semantic prototypes and further refines ambiguous boundary regions by integrating predictive entropy and foreground boundary responses.

This article develops a task-specific benign–malignant-aware segmentation framework for thyroid ultrasound images. Instead of relying solely on SegFormer as a conventional feature extractor, the proposed framework introduces prototype-supervised semantic calibration and uncertainty-guided boundary residual refinement into the segmentation process, enabling joint modeling of category discrimination and boundary correction. The overall architecture is illustrated in Figure 2.

Given an input ultrasound image $X\in R^{H\times W\times C}$ , the model first maps the original image into token representations through patch partition and linear embedding and then performs hierarchical feature extraction through multi-stage patch merging and SegFormer blocks. Each SegFormer block consists of a normalization layer, a multi-head self-attention module, and a feed-forward network, while residual connections are used to maintain stable feature propagation. In this way, multi-scale representations containing both local texture details and global contextual information can be obtained. The overall encoding process can be formulated as follows:

$$\left\{F_{1},F_{2},F_{3},F_{4}\right\}=E_{\text{SegFormer}}\left(X\right)=B_{4}◦M_{3}◦B_{3}◦M_{2}◦B_{2}◦M_{1}◦B_{1}◦P\left(X\right),$$ (1)

As shown in Formula 1, where P(⋅) denotes the initial patch partition and linear embedding operation, $B_{i}\left(\cdot \right)$ denotes the i th SegFormer encoding stage, $M_{i}\left(\cdot \right)$ denotes the patch merging operation between adjacent stages, and $F_{i}$ denotes the feature representation output by the i th hierarchical level. Through this hierarchical encoding strategy, the model can preserve thyroid nodule boundaries, echo textures, and local morphological information in shallow layers, while modeling lesion-related semantic context under a larger receptive field in deeper layers. These multi-level features provide fundamental feature support for subsequent benign–malignant-aware segmentation.

After obtaining hierarchical semantic features, the model further introduces an adaptive semantic prototype calibration module to establish explicit category prototypes for the background, benign nodules, and malignant nodules. Prototype-guided responses are generated based on the similarity between pixel features and category prototypes, thereby enhancing semantic separability across regions. Meanwhile, the class-conditional boundary refinement module integrates SegFormer-based predictions, adaptive semantic prototype calibration (ASPC) prototype responses, predictive entropy uncertainty, and foreground boundary responses to perform class-aware residual correction for ambiguous boundary regions and benign–malignant confusing regions. The overall prediction process can be formulated as follows:

$$L_{\text{base}}=H\left({\left\{F_{i}\right\}}_{i=1}^{4}\right),\;L_{\text{proto}}=A_{\text{ASPC}}\left({\left\{F_{i}\right\}}_{i=1}^{4}\right),$$ (2)

$$\hat{Y}=\underset{c}{arg\;max}R_{\text{CCBR}}\left(L_{\text{base}},L_{\text{proto}},U,G\right).$$ (3)

As shown in Formulas 2 and 3 where H(⋅) denotes the basic segmentation prediction head, $L_{\text{base}}$ denotes the basic segmentation logits generated from SegFormer features, $A_{\text{ASPC}}\left(\cdot \right)$ denotes the adaptive semantic prototype calibration process, $L_{\text{proto}}$ denotes the prototype-calibrated logits, U denotes the pixel-level uncertainty response computed from the predictive distribution, G denotes the boundary response extracted from the foreground probability gradient, $R_{\text{CCBR}}\left(\cdot \right)$ denotes the class-conditional boundary refinement operation, and $\hat{Y}$ denotes the final three-class segmentation result. Instead of performing ordinary foreground segmentation only for nodule regions, the proposed architecture incorporates benign and malignant nodules into a unified semantic segmentation space. Therefore, the model simultaneously achieves lesion localization, boundary refinement, and discrimination of regions at high risk of malignancy, thereby improving the clinical interpretability and task specificity of thyroid ultrasound image analysis.

### 3.2 Adaptive semantic prototype calibration, ASPC

**FIGURE 3:**
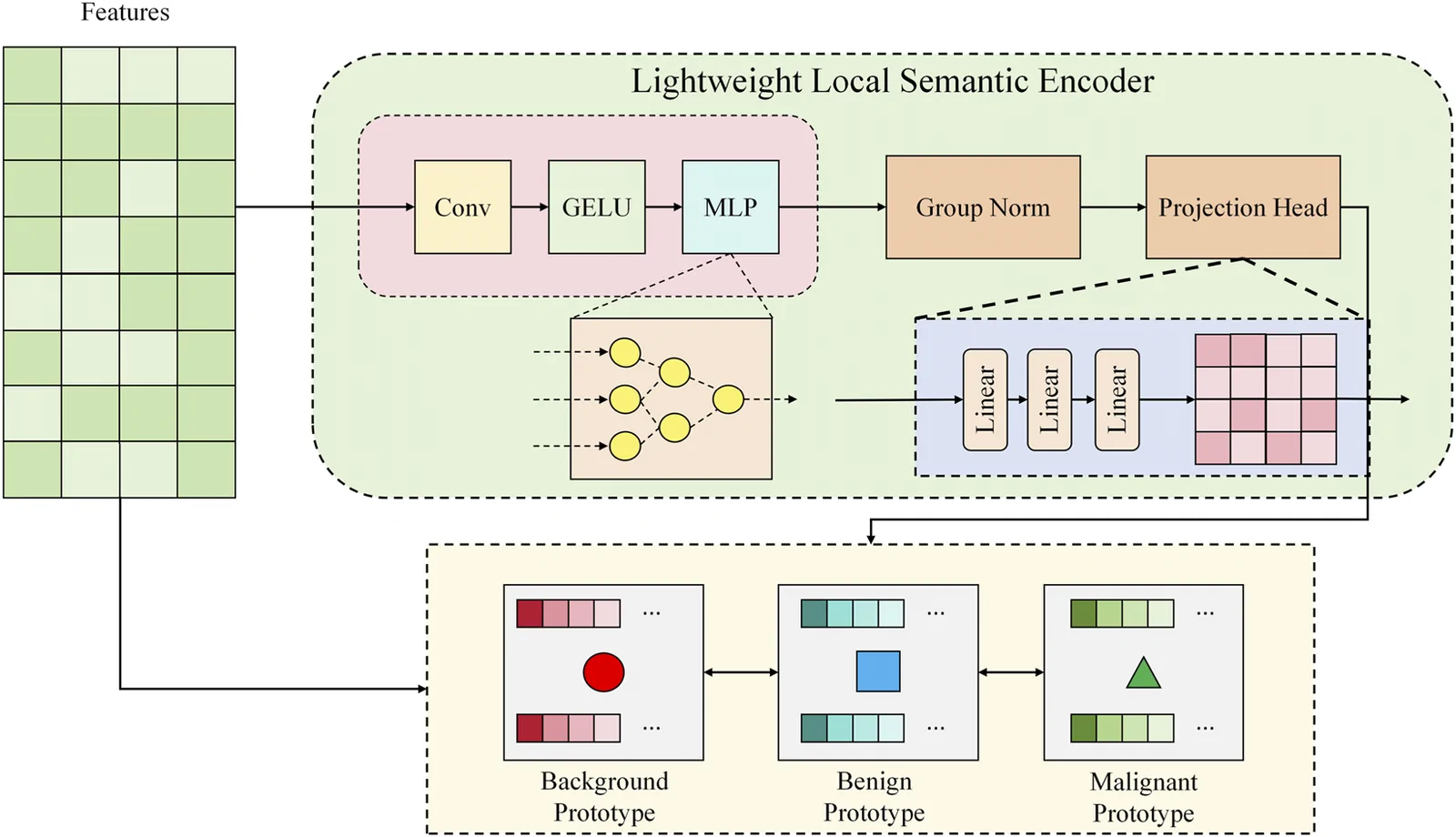
The module first performs nonlinear mapping and projection on the input features through a lightweight local semantic encoder, thereby obtaining pixel-level semantic representations with stronger category discriminability. Subsequently, the model constructs category prototypes for the background, benign nodules, and malignant nodules, respectively, and achieves category calibration through semantic matching between pixel features and prototypes, thus enhancing the separability of benign and malignant nodule regions.

After multi-scale feature extraction, the decoder features still face a key problem: pixels from benign and malignant nodules may have similar local textures and weak boundary contrast, which makes direct classification by a conventional segmentation head unstable. To reduce this category ambiguity, ASPC introduces a prototype-supervised calibration process into the prediction stage. Specifically, category prototypes are assigned to the background, benign nodule, and malignant nodule classes, and each pixel feature is compared against these prototypes in the semantic embedding space. The resulting prototype-calibrated responses provide an additional category-level reference for the final prediction, rather than relying only on local pixel-wise logits. In this way, ASPC strengthens the correspondence between class labels and semantic feature centers and helps the model distinguish benign and malignant regions under visually similar ultrasound patterns. The architecture of this module is shown in Figure 3.

First, features from different stages of the encoder are mapped to the same spatial scale, and the input feature of ASPC is formed through a feature fusion function:

$$F_{\text{a}}=\phi_{\text{fuse}}\left(\text{Concat }\left(\text{Up }\left(F_{1}\right),\text{Up }\left(F_{2}\right),\text{Up }\left(F_{3}\right),\text{Up }\left(F_{4}\right)\right)\right),$$ (4)

As shown in Formula 4, where Up (⋅) denotes the scale alignment operation, Concat (⋅) denotes concatenation along the channel dimension, $\phi_{\text{fuse}}\left(\cdot \right)$ denotes the feature fusion mapping, and $F_{\text{a}}$ denotes the ASPC input feature corresponding to ${\left\{F_{i}\right\}}_{i=1}^{4}$ in the overall architecture described above.

To further obtain pixel-level semantic representations suitable for prototype matching, ASPC adopts a lightweight local semantic encoder to perform local context enhancement and channel projection on $F_{\text{a}}$ . This encoder consists of convolutional mapping, nonlinear activation, a locally aware multi-layer perceptron (MLP), group normalization, and a projection head. Its function is to strengthen the representation ability of local textures, boundary neighborhoods, and internal echo patterns of nodules without significantly increasing computational cost. This process can be formulated as:

$$Z_{\text{a}}=\phi_{\text{proj}}\left(\text{GN }\left(\text{MLP }\left(\sigma \left({\text{Conv }}_{3\times 3}\left(F_{\text{a}}\right)\right)\right)\right)\right),$$ (5)

As shown in Formula 5, where σ(⋅) denotes the Gaussian error linear unit (GELU) activation function, GN (⋅) denotes group normalization, $\phi_{\text{proj}}\left(\cdot \right)$ denotes the projection head, and $Z_{\text{a}}\in R^{B\times D\times H\times W}$ denotes the pixel-level semantic embedding feature output by ASPC. To enable stable matching between this feature and the category prototypes, channel-wise normalization is further applied to the pixel features:

$${\hat{Z}}_{\text{a}}\left(i,j\right)=\frac{Z_{\text{a}}\left(i,j\right)}{{\left\|Z_{\text{a}}\left(i,j\right)\right\|}_{2}+\epsilon },$$ (6)

As shown in Formula 6, where (i,j) denotes the spatial position and ϵ is a very small constant used to avoid numerical instability. After normalization, the feature magnitude differences across different pixel positions are suppressed, and category discrimination is mainly determined by directional similarity, which is beneficial for establishing a more stable semantic prototype matching relationship.

ASPC constructs a learnable prototype library for three semantic regions: background, benign nodules, and malignant nodules. The prototype library is denoted as $P\in R^{K\times D}$ , where K=3 , and each prototype vector represents the central semantic reference of one category in the semantic embedding space. The prototypes are static learnable parameters initialized during model construction and updated by back-propagation during training, rather than being dynamically updated by a memory bank or moving-average strategy. The prototype library is defined as follows:

$$P=\left[p_{0};p_{1};p_{2}\right],\;p_{c}\in R^{D},\;c\in \left\{0,1,2\right\},$$ (7)

As shown in Formula 7, where $p_{0}$ , $p_{1}$ , and $p_{2}$ denote the background prototype, benign nodule prototype, and malignant nodule prototype, respectively. To explicitly constrain these prototypes to their corresponding semantic categories, the prototype-calibrated logits are supervised by the class-conditional label map through the auxiliary prototype loss introduced in the training objective. To remain consistent with pixel-level semantic features, the category prototypes are also normalized by $L_{2}$ normalization:

$${\hat{p}}_{c}=\frac{p_{c}}{{\left\|p_{c}\right\|}_{2}+\epsilon }.$$ (8)

As shown in Formula 8, in this way, ASPC transforms the conventional pixel classification process into a matching process between pixel semantic features and category prototypes. Therefore, each pixel is not only classified based on local convolutional responses or decoder outputs but is also explicitly assigned to the category-level semantic centers of the background, benign nodules, and malignant nodules. In this context, “adaptive” refers to the joint optimization of category prototypes, learnable temperature scaling, category bias, and pixel–prototype similarity responses, which allows the prototype response intensity to be adjusted during training.

Finally, ASPC generates prototype-calibrated logits by computing cosine similarities between pixel features and normalized category prototypes and introduces a learnable temperature factor and category bias to adaptively adjust the response intensities across categories. For the prototype response of category c at spatial position (i,j) , the calculation is defined as follows:

$$L_{\text{proto}}^{c}\left(i,j\right)=s\cdot \left({\hat{Z}}_{\text{a}}{\left(i,j\right)}^{⊤}{\hat{p}}_{c}\right)+b_{c},$$ (9)

As shown in Formula 9, where s denotes the learnable temperature scaling factor, $b_{c}$ denotes the learnable bias term of the c th category, and $L_{\text{proto}}\in R^{B\times K\times H\times W}$ denotes the prototype-calibrated logits generated by ASPC. Thus, $L_{\text{proto}}=A_{\text{ASPC}}\left({\left\{F_{i}\right\}}_{i=1}^{4}\right)$ in the overall architecture described above is concretely implemented. This response is then jointly fed into the class-conditional boundary refinement module together with the SegFormer basic prediction $L_{\text{base}}$ , the predictive entropy uncertainty U , and the foreground boundary response G . As a result, the subsequent boundary correction depends not only on spatial contour information but also on the semantic prototypes of benign and malignant categories, thereby improving category consistency and lesion-region discrimination in three-class thyroid nodule segmentation.

### 3.3 Class-conditional boundary refinement, CCBR

**FIGURE 4:**
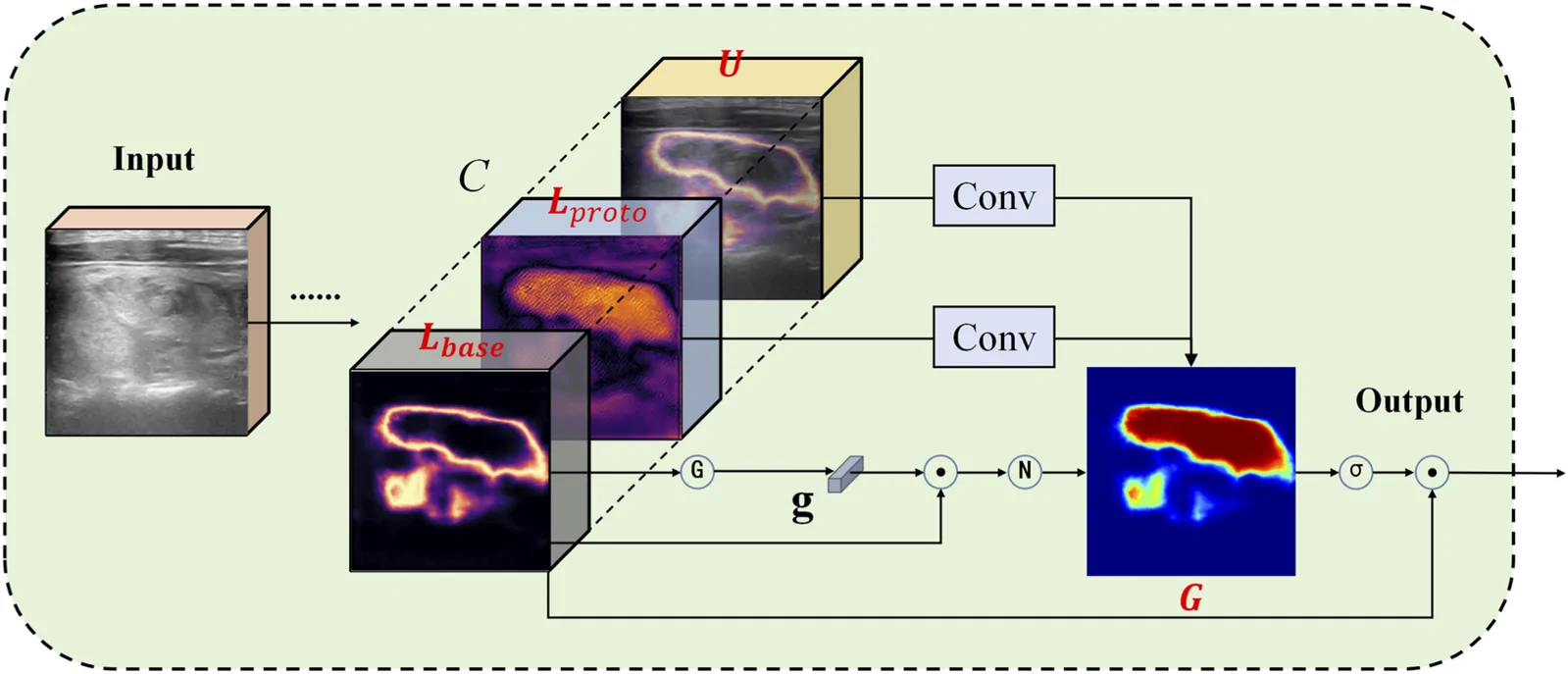
The overall process of the category condition boundary refinement module is as follows: This module integrates the basic prediction response, ASPC prototype response, prediction uncertainty, and foreground boundary response to adaptively correct the fuzzy boundary of thyroid nodules and the easily confused areas of benign and malignant.

After prototype-calibrated responses are obtained, CCBR is introduced to further refine regions where category prediction and boundary localization are both uncertain. The key design of CCBR is not to perform independent edge enhancement, but to convert semantic confidence, prototype response, and foreground transition information into a unified residual correction process. Specifically, the basic logits $L_{\text{base}}$ provide the original segmentation response, while the prototype logits $L_{\text{proto}}$ provide category-level semantic guidance. Meanwhile, the predictive uncertainty U highlights ambiguous pixels, and the foreground boundary response G indicates sharp transitions between nodule and background regions. By jointly modeling these complementary cues, CCBR adaptively adjusts the correction intensity in low-contrast boundaries, weak-texture regions, and benign–malignant confusing areas, thereby improving boundary consistency without relying on a single hand-crafted edge cue. The architecture of this module is shown in Figure 4.

First, the basic prediction logits are converted into a category probability distribution, and the pixel-level category uncertainty is further characterized by predictive entropy:

$$P=\text{Softmax }\left(L_{\text{base}}\right),\;P\in R^{B\times K\times H\times W},$$ (10)

$$U_{i,j}=-\overset{K}{\underset{k=1}{\sum }}P_{k,i,j}\log\left(P_{k,i,j}+\epsilon \right),\;U\in R^{B\times 1\times H\times W},$$ (11)

As shown in Formulas 10 and 11, where K=3 denotes the three categories, namely, background, benign nodules, and malignant nodules, and ϵ is a numerical stability term. A higher value of U indicates stronger uncertainty in category discrimination at the corresponding position, which usually appears in low-contrast boundaries, regions with heterogeneous internal echoes, or areas with benign–malignant category confusion. Therefore, it can serve as an important guidance signal for subsequent boundary refinement. During training, U is used as a detached guidance map before being fed into the refinement branch, so that the entropy response guides boundary correction without introducing additional gradient paths through the uncertainty computation.

To further explicitly model nodule edge variations, CCBR aggregates the benign nodule probability and malignant nodule probability into a foreground probability map and extracts the foreground boundary response based on spatial gradients. This process can weaken the interference of background regions in boundary estimation, enabling the model to focus more on the transition zone between the nodule region and surrounding tissues:

$$P_{\text{fg}}=P_{1}+P_{2},$$ (12)

$$G=\text{Norm }\left(\sqrt{{\left(S_{x}\left(P_{\text{fg}}\right)\right)}^{2}+{\left(S_{y}\left(P_{\text{fg}}\right)\right)}^{2}}\right),\;G\in R^{B\times 1\times H\times W},$$ (13)

As shown in Formulas 12 and 13, where $S_{x}\left(\cdot \right)$ and $S_{y}\left(\cdot \right)$ denote the spatial gradient operators in the horizontal and vertical directions, respectively, and Norm (⋅) denotes the normalization operation. In the implementation, $S_{x}\left(\cdot \right)$ and $S_{y}\left(\cdot \right)$ are fixed Sobel operators rather than learnable convolution kernels. Norm (⋅) is implemented as min–max normalization within each feature map to rescale the boundary response into the range of [0, 1]. Similar to U , G is also detached before concatenation with other responses and is used only as an explicit boundary guidance signal. Through this design, G can highlight the positions where the foreground probability changes most sharply, thereby providing an explicit contour response for the model. Meanwhile, U reflects the model uncertainty in pixel-level classification. These two signals complement the basic prediction from the perspectives of boundary structure and category confidence.

After constructing the multi-source responses, CCBR concatenates $L_{\text{base}}$ , $L_{\text{proto}}$ , U , and G along the channel dimension and then generates the boundary refinement response through a lightweight convolutional branch. At the same time, the module generates a spatial modulation map A according to the fused features to adaptively control the correction intensity in different regions, so that boundary refinement is concentrated on regions with high uncertainty and strong boundary responses:

$$Z=\text{Concat }\left(L_{\text{base}},L_{\text{proto}},U,G\right),\;Z\in R^{B\times \left(2K+2\right)\times H\times W},$$ (14)

$$A=\sigma \left(N\left(\phi_{\text{g}}\left(Z\right)\right)\right),\;R=\phi_{\text{r}}\left(Z\right),$$ (15)

As shown in Formulas 14 and 15, where $\phi_{\text{g}}\left(\cdot \right)$ denotes the spatial modulation branch composed of convolutional mappings, N(⋅) denotes the normalization operation, σ(⋅) denotes the sigmoid activation function, A denotes the adaptive response map for boundary refinement, $\phi_{\text{r}}\left(\cdot \right)$ denotes the residual correction branch, and R denotes the category-related boundary residual. Finally, CCBR fuses the basic prediction, prototype response, and modulated residual correction to obtain the final segmentation result:

$$L_{\text{final}}=L_{\text{base}}+\alpha L_{\text{proto}}+A⊙R,\;\hat{Y}=\underset{k}{arg\;max}L_{\text{final}}^{\left(k\right)},$$ (16)

As shown in Formula 16, where α denotes the fusion weight of the prototype response, ⊙ denotes element-wise modulation, and $L_{\text{final}}$ denotes the final logits after boundary refinement. Through this mechanism, CCBR unifies semantic prototypes, predictive entropy, and foreground boundary information into a single refinement process, enabling the model to perform more stable pixel-level correction on thyroid nodule boundaries and benign–malignant confusing regions while maintaining overall semantic discriminability.

### 3.4 Loss function and training objective

After the CCBR module produces the final logits $L_{\text{final}}$ , the model is optimized using the three-class class-conditional nodule label map Y , including background, benign nodule, and malignant nodule. Considering the strong imbalance between background pixels and nodule pixels, especially the limited number of malignant pixels, the main segmentation objective combines weighted cross-entropy loss and Dice loss on $L_{\text{final}}$ :

$$L_{\text{seg}}=L_{\text{wce}}\left(L_{\text{final}},Y\right)+\lambda_{\text{dice}}L_{\text{dice}}\left(L_{\text{final}},Y\right),$$ (17)

As shown in Formula 17, where $L_{\text{wce}}$ denotes the weighted cross-entropy loss with class weights computed from the pixel frequency of the training set and $L_{\text{dice}}$ is used to improve region-level overlap consistency. In all experiments, $\lambda_{\text{dice}}$ is set to 1.0.

To make the category prototypes explicitly correspond to the background, benign, and malignant categories, the prototype-calibrated logits $L_{\text{proto}}$ are additionally supervised by the same label map. As shown in Formula 18,

$$L_{\text{proto}}=L_{\text{wce}}\left(L_{\text{proto}},Y\right).$$ (18)

Therefore, the total training objective is defined as follows:

$$L_{\text{total}}=L_{\text{seg}}+\lambda_{\text{proto}}L_{\text{proto}},$$ (19)

As shown in Formula 19, where $\lambda_{\text{proto}}$ is set to 0.5. The prototypes in ASPC are learnable parameters updated by back-propagation during training.

## 4 Experimental results and analysis

### 4.1 Experimental setup

The experiments in this study were conducted on a Linux server. Both model training and testing were implemented using the PyTorch deep learning framework, and the pretrained SegFormer weights were loaded using HuggingFace Transformers. The input thyroid ultrasound images were uniformly resized to 512×512 , and the segmentation task was formulated as a three-class semantic segmentation problem, including background, benign nodules, and malignant nodules. During training, the AdamW optimizer was used for parameter updates, with the initial learning rate set to $6\times 10^{-5}$ , and the weight decay coefficient set to $1\times 10^{-4}$ . A cosine annealing strategy was used to adjust the learning rate. To ensure experimental stability and reproducibility, all experiments were repeated three times with different random seeds, and the reported mean and standard deviation were calculated from these three independent runs. The number of training epochs was set to 200, and the batch size was set to 8. Mixed-precision training was also enabled to improve computational efficiency. For all comparison baselines, the same data split, input size, training epochs, augmentation strategy, loss setting, validation-based model selection rule, and three-class output adaptation were used, and the baseline models were implemented based on public code or reproduced according to the original articles. Model weights were saved every 10 epochs, and the checkpoint with the best validation mIoU was selected as the final model for testing. The test set was used only for the final performance evaluation. The main experimental environment and training hyperparameters are shown in Table 2.

**TABLE 2:** Experimental environment and main training hyperparameter settings.

Item	Setting
Operating system	Linux
Graphics card	NVIDIA A100 GPU
Deep learning framework	PyTorch
Model implementation tool	HuggingFace Transformers
Backbone network	SegFormer-B0
Pretrained weights	ADE20K pretrained weights
Input image size	512×512
Number of classes	3, background/benign/malignant
Training epochs	200
Batch size	8
Optimizer	AdamW
Initial learning rate	$6\times 10^{-5}$
Weight decay	$1\times 10^{-4}$
Learning rate scheduler	Cosine annealing
Minimum learning rate	$1\times 10^{-6}$
Validation split	0.2
Mixed-precision training	Enabled
Gradient clipping threshold	1.0
Model saving strategy	Save weights every 10 epochs
Prototype dimension D	64
Prototype fusion weight α	0.5
Inference time	18.72 m/image

### 4.2 Evaluation metric

To comprehensively evaluate the performance of the model in the three-class segmentation task for thyroid ultrasound images, Pixel Accuracy, mIoU, mDice, and mPrecision are adopted as the main evaluation metrics. Let the number of classes be K=3 , corresponding to background, benign, and malignant, respectively. $TP_{c}$ , $FP_{c}$ , and $FN_{c}$ denote the numbers of true positive, false positive, and false negative pixels for the c th class, respectively.

Pixel Accuracy is used to measure the proportion of correctly classified pixels among all pixels, reflecting the overall accuracy of pixel-level prediction. It is calculated as:

$$Pixel\;Accuracy=\frac{\sum_{c=1}^{K}TP_{c}}{\sum_{c=1}^{K}\left(TP_{c}+FP_{c}\right)}.$$ (20)

As shown in Formula 20, a higher value of this metric indicates more accurate classification results across the entire image. However, under class imbalance, it may be affected by the large proportion of background pixels.

mIoU, a commonly used comprehensive evaluation metric in semantic segmentation tasks, measures the average overlap between the predicted and ground-truth regions. It is calculated as follows:

$$mIoU=\frac{1}{K}\overset{K}{\underset{c=1}{\sum }}\frac{TP_{c}}{TP_{c}+FP_{c}+FN_{c}}.$$ (21)

As shown in Formula 21, this metric accounts for both missed and false segmentation, making it more suitable for evaluating the segmentation quality between thyroid nodule regions and background regions.

mDice is used to measure the similarity between predicted and ground-truth regions and is especially suitable for lesion segmentation in medical images where lesion regions occupy a relatively small proportion. It is calculated as follows:

$$mDice=\frac{1}{K}\overset{K}{\underset{c=1}{\sum }}\frac{2TP_{c}}{2TP_{c}+FP_{c}+FN_{c}}.$$ (22)

As shown in Formula 22, a higher value of this metric indicates stronger consistency between the predicted and the ground-truth annotated regions and can more intuitively reflect the segmentation completeness of nodule regions.

mPrecision is used to measure the proportion of pixels that truly belong to a certain class among all pixels predicted as that class, reflecting the model’s ability to control false-positive regions. It is calculated as follows:

$$mPrecision=\frac{1}{K}\overset{K}{\underset{c=1}{\sum }}\frac{TP_{c}}{TP_{c}+FP_{c}}.$$ (23)

As shown in Formula 23, a higher value of this metric indicates fewer false-positive pixels when the model predicts benign nodules, malignant nodules, and background regions, suggesting greater reliability of the segmentation results.

HD95 is used to evaluate the boundary distance between the predicted segmentation region and the ground-truth annotation region. In this study, HD95 is calculated on the foreground nodule mask, where benign and malignant nodule pixels are merged into a single foreground region. Let P and G denote the boundary point sets of the predicted foreground mask and the ground-truth foreground mask, respectively. It is calculated as follows:

$$HD95=\max(Q_{95}\left(\left\{\underset{g\in G}{\min}\|p-g{\|}_{2}|p\in P\right\}\right),Q_{95}\left(\left\{\underset{p\in P}{\min}\|g-p{\|}_{2}|g\in G\right\}\right)),$$ (24)

As shown in Formula 24, where $Q_{95}\left(\cdot \right)$ denotes the 95th percentile of the point-to-set distance distribution. A lower HD95 value indicates smaller boundary deviation and better contour consistency between the predicted nodule region and the ground-truth annotation.

In addition to the main metrics above, this study reports foreground nodule Dice and intersection over union (IoU), per-class Dice and IoU for benign and malignant nodules, and the sensitivity and specificity of malignancy detection to provide a more detailed evaluation of nodule localization and benign–malignant discrimination. For foreground evaluation, benign and malignant nodule pixels are merged into a single nodule region. For per-class Dice and IoU, classes absent from both the prediction and the ground truth are excluded from the corresponding image-level average to avoid undefined 0/0 cases, and the results are then averaged across images or cases. Sensitivity and specificity are used with precision to evaluate the performance of malignant detection and the potential miss rate of malignant nodules.

### 4.3 Experimental results compared with other models

To comprehensively verify the effectiveness of the proposed method in the benign–malignant-aware segmentation task of thyroid nodules, this study compares it with several representative medical image segmentation models and general semantic segmentation models. The compared methods include classical convolutional segmentation networks, atrous convolution and pyramid context modeling methods, high-resolution feature-preserving models, real-time segmentation models, and Transformer-based segmentation frameworks, thereby enabling a comprehensive evaluation of model performance across different network structure paradigms. All comparison models are trained and tested under the same data split, input size, and evaluation metric settings to ensure the fairness and comparability of the experimental results. The following tables present quantitative comparisons of different methods on the TN3K dataset and the external clinical validation dataset. First, the experimental results on the public TN3K dataset are presented, as shown in Table 3.

**TABLE 3:** Comparison results of different segmentation models on the TN3K dataset.

Method	PA	mIoU	mDice	mPrecision	HD95 ↓
U-Net (Ronneberger et al., 2015)	0.8536±0.0148	0.4279±0.0187	0.5612±0.0209	0.6185±0.0234	61.84±4.76
DeepLabv3+ (Chen et al., 2018)	0.8662±0.0161	0.4518±0.0216	0.5874±0.0192	0.6429±0.0268	58.37±5.12
PSPNet (Zhao et al., 2017)	0.8875±0.0107	0.4973±0.0179	0.6326±0.0145	0.6962±0.0217	52.49±3.95
TNSNet (Sun et al., 2022)	0.9048±0.0116	0.5527±0.0184	0.6769±0.0175	0.7286±0.0228	46.73±3.68
HRNet (Sun et al., 2019)	0.9124±0.0089	0.5668±0.0134	0.6897±0.0129	0.7445±0.0158	43.58±3.21
PIDNet (Xu et al., 2023)	0.9017±0.0122	0.5386±0.0196	0.6663±0.0181	0.7194±0.0241	48.26±4.14
BPAT-UNet (Bi et al., 2023)	0.9166±0.0101	0.5869±0.0167	0.7048±0.0156	0.7532±0.0204	40.69±3.47
Mask2Former (Cheng et al., 2022)	0.9208±0.0076	0.6025±0.0128	0.7141±0.0109	0.7796±0.0173	36.82±2.85
Segmenter (Strudel et al., 2021)	0.9063±0.0115	0.5731±0.0162	0.6978±0.0147	0.7560±0.0205	42.17±3.64
SegMan (Fu et al., 2025)	0.9179±0.0093	0.5914±0.0151	0.7075±0.0133	0.7662±0.0188	38.54±3.09
PraNet-V2 (Hu et al., 2026)	0.9102±0.0129	0.5489±0.0214	0.6735±0.0198	0.7357±0.0250	47.62±4.31
SECNet (Li et al., 2025)	0.9236±0.0081	0.6108±0.0119	0.7224±0.0112	0.7891±0.0165	35.76±2.74
TransDeep (Chai et al., 2025)	0.9147±0.0104	0.5826±0.0143	0.7012±0.0168	0.7604±0.0199	41.36±3.52
CLAC-Net (Feng et al., 2025)	0.9271±0.0074	0.6243±0.0106	0.7349±0.0097	0.7979±0.0142	33.94±2.41
Ours	0.9449±0.0048	0.6427±0.0079	0.7517±0.0066	0.8147±0.0113	28.57±2.86

As shown in Table 3, the proposed method achieves the highest point estimates across all four metrics on the TN3K dataset, with PA, mIoU, mDice, and mPrecision reaching 0.9449, 0.6427, 0.7517, and 0.8147, respectively. These results indicate that the proposed method not only maintains high overall pixel-level classification accuracy but also achieves more stable performance across nodule-region overlap, segmentation consistency, and false-positive control. Compared with traditional convolutional segmentation models such as U-Net, DeepLabv3+, and PSPNet, the proposed method shows numerical improvements, suggesting that simply relying on local convolutional features is insufficient to fully address the segmentation challenges posed by low contrast, weak boundaries, and complex echo textures in thyroid ultrasound images. Compared with stronger Transformer-based or hybrid models such as Mask2Former, Segmenter, SegMan, and CLAC-Net, the proposed method still yields higher point estimates on the reported metrics, indicating that relying only on global context modeling may not be sufficient to address the fine-grained differences between benign and malignant nodule regions.

As further shown in Table 4, the proposed method also yields higher point estimates for foreground nodule segmentation, benign and malignant class-specific segmentation, and malignancy detection. The numerically higher foreground Dice and IoU indicate better overall nodule localization, while the higher benign and malignant Dice/IoU values suggest stronger category-aware region discrimination. In addition, the numerically higher malignancy sensitivity and specificity suggest that the model may better balance malignant nodule recognition and false-positive control. Therefore, the detailed results provide additional evidence of the effectiveness of ASPC for category-level semantic calibration and of CCBR in uncertain boundary correction. Further experimental results using the supervised in-house clinical setting are presented in Table 5.

**TABLE 4:** Detailed segmentation and malignancy detection results of different models on the TN3K dataset.

Method	FG dice	FG IoU	Benign dice	Benign IoU	Malignant dice	Malignant IoU	Malignant Sens.	Malignant Spec.
U-Net	0.6463±0.0268	0.4774±0.0295	0.3916±0.0341	0.2261±0.0276	0.3275±0.0412	0.1826±0.0314	0.5167±0.0465	0.7869±0.0278
DeepLabv3+	0.6684±0.0247	0.5019±0.0321	0.4378±0.0308	0.2592±0.0294	0.3682±0.0397	0.2122±0.0346	0.5534±0.0428	0.8027±0.0311
PSPNet	0.7037±0.0215	0.5428±0.0256	0.4903±0.0279	0.3248±0.0253	0.4327±0.0334	0.2761±0.0306	0.5982±0.0369	0.8246±0.0235
TNSNet	0.7401±0.0197	0.5875±0.0228	0.5808±0.0246	0.4092±0.0231	0.5162±0.0315	0.3479±0.0286	0.6419±0.0342	0.8506±0.0208
HRNet	0.7552±0.0168	0.6067±0.0192	0.5958±0.0217	0.4243±0.0204	0.5413±0.0278	0.3711±0.0247	0.6635±0.0316	0.8647±0.0172
PIDNet	0.7384±0.0221	0.5853±0.0264	0.5587±0.0292	0.3876±0.0271	0.4953±0.0358	0.3292±0.0322	0.6368±0.0394	0.8479±0.0216
BPAT-UNet	0.7720±0.0169	0.6287±0.0195	0.6190±0.0214	0.4482±0.0203	0.5734±0.0276	0.4019±0.0258	0.6976±0.0317	0.8769±0.0186
Mask2Former	0.8012±0.0143	0.6684±0.0165	0.6380±0.0189	0.4684±0.0181	0.5956±0.0242	0.4241±0.0235	0.7186±0.0297	0.8915±0.0153
Segmenter	0.7681±0.0196	0.6235±0.0237	0.6099±0.0234	0.4387±0.0251	0.5450±0.0295	0.3746±0.0272	0.6819±0.0338	0.8696±0.0211
SegMan	0.7909±0.0172	0.6541±0.0213	0.6234±0.0207	0.4529±0.0226	0.5809±0.0261	0.4093±0.0254	0.7074±0.0306	0.8842±0.0193
PraNet-V2	0.7472±0.0245	0.5964±0.0288	0.5740±0.0305	0.4025±0.0309	0.5099±0.0372	0.3422±0.0338	0.6521±0.0416	0.8563±0.0257
SECNet	0.8115±0.0132	0.6828±0.0157	0.6510±0.0178	0.4826±0.0172	0.6032±0.0229	0.4318±0.0218	0.7314±0.0271	0.8987±0.0145
TransDeep	0.7797±0.0184	0.6389±0.0209	0.6141±0.0228	0.4431±0.0239	0.5670±0.0283	0.3957±0.0267	0.6956±0.0324	0.8781±0.0184
CLAC-Net	0.8269±0.0124	0.7049±0.0149	0.6700±0.0164	0.5038±0.0158	0.6189±0.0206	0.4481±0.0201	0.7489±0.0254	0.9075±0.0136
Ours	0.8449±0.0093	0.7314±0.0117	0.6908±0.0131	0.5276±0.0139	0.6436±0.0184	0.4745±0.0189	0.7798±0.0226	0.9172±0.0108

**TABLE 5:** Comparison results of different segmentation models on the in-house clinical dataset under the supervised case-level training and testing split. All models were trained on the in-house clinical training set and evaluated on the held-out in-house clinical test set, which differs from the external transfer and mixed-domain adaptation setting.

Method	PA	mIoU	mDice	mPrecision	HD95 ↓
U-Net	0.7634±0.0197	0.4218±0.0275	0.5486±0.0312	0.6079±0.0248	57.86±5.43
DeepLabv3+	0.7782±0.0215	0.4469±0.0251	0.5794±0.0298	0.6233±0.0287	53.72±6.18
PSPNet	0.8016±0.0152	0.4927±0.0189	0.6411±0.0244	0.6925±0.0206	46.35±4.27
TNSNet	0.8198±0.0139	0.5576±0.0176	0.6961±0.0208	0.7394±0.0187	39.84±3.91
HRNet	0.8239±0.0108	0.5483±0.0147	0.6886±0.0181	0.7480±0.0154	41.26±3.36
PIDNet	0.8088±0.0186	0.5314±0.0202	0.6702±0.0267	0.7213±0.0179	44.58±4.76
BPAT-UNet	0.8311±0.0125	0.5864±0.0168	0.7087±0.0189	0.7898±0.0146	34.96±3.68
Mask2Former	0.8336±0.0119	0.5967±0.0135	0.7158±0.0164	0.7811±0.0128	32.47±2.94
Segmenter	0.8145±0.0141	0.5679±0.0180	0.7042±0.0219	0.7426±0.0201	37.15±4.02
SegMan	0.8294±0.0136	0.5832±0.0156	0.7065±0.0195	0.7719±0.0149	35.72±3.15
PraNet-V2	0.8207±0.0169	0.5526±0.0223	0.6974±0.0176	0.7587±0.0226	40.63±5.06
SECNet	0.8355±0.0107	0.5891±0.0172	0.7137±0.0142	0.7768±0.0167	33.18±2.51
TransDeep	0.8271±0.0124	0.5748±0.0193	0.7109±0.0188	0.7640±0.0191	36.81±4.35
CLAC-Net	0.8322±0.0151	0.5934±0.0126	0.7169±0.0157	0.7835±0.0131	31.54±2.39
Ours	0.8521±0.0094	0.6124±0.0118	0.7328±0.0122	0.7996±0.0105	27.16±3.23

As shown in Table 5, under the supervised case-level, in-house clinical training, and testing split, the overall performance of each model shows a more obvious decline compared with that on the public dataset. This suggests that real clinical ultrasound images exhibit stronger domain differences and higher complexity in terms of imaging equipment, scanning angles, nodule size, boundary clarity, and tissue echo distribution. Under these conditions, traditional convolutional models such as U-Net, DeepLabv3+, and PSPNet show relatively limited performance, suggesting that local texture modeling and fixed-scale context aggregation may be insufficient to fully adapt to weak boundaries and heterogeneous noise in clinical images. Although methods such as Mask2Former, CLAC-Net, and SECNet achieve competitive results, they yield lower point estimates than the proposed method across mIoU, mDice, and mPrecision. The proposed method achieves PA, mIoU, mDice, and mPrecision values of 0.8521, 0.6124, 0.7328, and 0.7996, respectively, suggesting improved clinical-domain segmentation performance under supervised in-house adaptation rather than direct external transfer.

Table 6 further demonstrates the effectiveness of the proposed method in foreground nodule delineation, class-specific region discrimination, and malignancy-related recognition. Compared with other methods, the proposed model achieves higher point estimates for foreground Dice and IoU, indicating potentially more complete and accurate localization of nodule regions under complex clinical ultrasound conditions. In terms of benign and malignant class-specific segmentation, the proposed method also achieves higher Dice and IoU scores, suggesting that the semantic prototype calibration mechanism enhances the distinction between different nodule categories, while the boundary refinement strategy contributes to more stable contour correction. Moreover, the numerically higher malignancy sensitivity suggests a potential reduction in the risk of missing malignant nodules, and the higher specificity suggests improved control of false malignant predictions. These results indicate that the proposed method may provide more balanced performance in nodule segmentation, benign–malignant region discrimination, and malignancy detection.

**TABLE 6:** Detailed pixel-level segmentation and malignant-class discrimination results of different models on the in-house clinical dataset under the supervised case-level training and testing split. All models were trained on the in-house clinical training set and evaluated on the held-out in-house clinical test set, which differs from the external transfer and mixed-domain adaptation setting.

Method	FG dice	FG IoU	Benign dice	Benign IoU	Malignant dice	Malignant IoU	Malig. Sens.	Malig. Spec.
U-Net	0.6328±0.0317	0.4629±0.0354	0.4074±0.0392	0.2558±0.0326	0.3258±0.0475	0.1946±0.0361	0.4873±0.0528	0.7425±0.0346
DeepLabv3+	0.6542±0.0295	0.4861±0.0332	0.4538±0.0367	0.2935±0.0314	0.3622±0.0446	0.2212±0.0385	0.5236±0.0494	0.7598±0.0372
PSPNet	0.7002±0.0246	0.5387±0.0278	0.5258±0.0315	0.3567±0.0286	0.4367±0.0392	0.2794±0.0347	0.5748±0.0431	0.7916±0.0294
TNSNet	0.7572±0.0224	0.6092±0.0257	0.6096±0.0261	0.4385±0.0248	0.5308±0.0356	0.3613±0.0314	0.6605±0.0417	0.8471±0.0239
HRNet	0.7437±0.0189	0.5918±0.0217	0.5963±0.0246	0.4248±0.0221	0.5241±0.0328	0.3551±0.0283	0.6429±0.0368	0.8337±0.0215
PIDNet	0.7285±0.0264	0.5725±0.0301	0.5701±0.0334	0.3987±0.0312	0.5047±0.0416	0.3375±0.0359	0.6112±0.0457	0.8174±0.0268
BPAT-UNet	0.7774±0.0198	0.6355±0.0231	0.6520±0.0234	0.4837±0.0215	0.5709±0.0318	0.3995±0.0287	0.7008±0.0369	0.8752±0.0206
Mask2Former	0.7814±0.0175	0.6412±0.0208	0.6592±0.0213	0.4916±0.0194	0.5871±0.0287	0.4155±0.0262	0.7025±0.0335	0.8659±0.0186
Segmenter	0.7605±0.0232	0.6136±0.0267	0.6256±0.0271	0.4552±0.0259	0.5470±0.0349	0.3765±0.0318	0.6684±0.0396	0.8443±0.0247
SegMan	0.7756±0.0211	0.6328±0.0249	0.6463±0.0252	0.4774±0.0235	0.5641±0.0315	0.3932±0.0294	0.6896±0.0351	0.8581±0.0209
PraNet-V2	0.7492±0.0278	0.5989±0.0325	0.6040±0.0346	0.4327±0.0327	0.5306±0.0403	0.3611±0.0364	0.6517±0.0462	0.8365±0.0289
SECNet	0.7783±0.0194	0.6371±0.0226	0.6509±0.0237	0.4825±0.0218	0.5753±0.0304	0.4038±0.0275	0.7091±0.0342	0.8726±0.0197
TransDeep	0.7682±0.0226	0.6237±0.0258	0.6355±0.0264	0.4658±0.0247	0.5545±0.0337	0.3836±0.0306	0.6758±0.0379	0.8529±0.0235
CLAC-Net	0.7856±0.0182	0.6468±0.0214	0.6561±0.0225	0.4882±0.0206	0.5795±0.0291	0.4080±0.0269	0.7164±0.0316	0.8793±0.0178
Ours	0.8015±0.0148	0.6685±0.0185	0.6797±0.0189	0.5148±0.0176	0.5978±0.0265	0.4264±0.0252	0.7469±0.0294	0.8897±0.0152

### 4.4 Ablation test results

To further verify the actual contributions of the two core modules, ASPC and CCBR, to model performance improvement, this study designs ablation experiments under the same experimental settings. It progressively removes or adds the corresponding modules for comparative analysis. Through this experiment, the roles of semantic prototype calibration and class-conditional boundary refinement in the benign–malignant-aware segmentation task of thyroid nodules can be more clearly evaluated. Table 7 presents the quantitative results of different model variants on the TN3K dataset and the external clinical validation dataset.

**TABLE 7:** Ablation test results on the TN3K dataset and the supervised case-level, in-house clinical setting.

Dataset	Method	PA	mIoU	mDice	mPrecision	HD95 ↓
TN3K	Baseline	0.9128±0.0069	0.5846±0.0107	0.6985±0.0083	0.7589±0.0146	39.84±3.27
	+ASPC	0.9279±0.0081	0.6113±0.0094	0.7221±0.0112	0.7980±0.0108	34.62±2.81
	+CCBR	0.9354±0.0055	0.6266±0.0131	0.7364±0.0074	0.7842±0.0153	31.16±2.43
	Ours	0.9449±0.0048	0.6427±0.0079	0.7517±0.0066	0.8147±0.0113	28.57±2.86
Supervised in-house clinical setting	Baseline	0.8136±0.0128	0.5532±0.0174	0.6741±0.0149	0.7395±0.0186	33.68±3.74
	+ASPC	0.8358±0.0111	0.5843±0.0139	0.7065±0.0167	0.7816±0.0134	31.94±3.12
	+CCBR	0.8294±0.0147	0.5908±0.0178	0.7160±0.0116	0.7663±0.0159	27.71±2.95
	Ours	0.8521±0.0094	0.6124±0.0118	0.7328±0.0122	0.7996±0.0105	27.16±3.23

**FIGURE 5:**
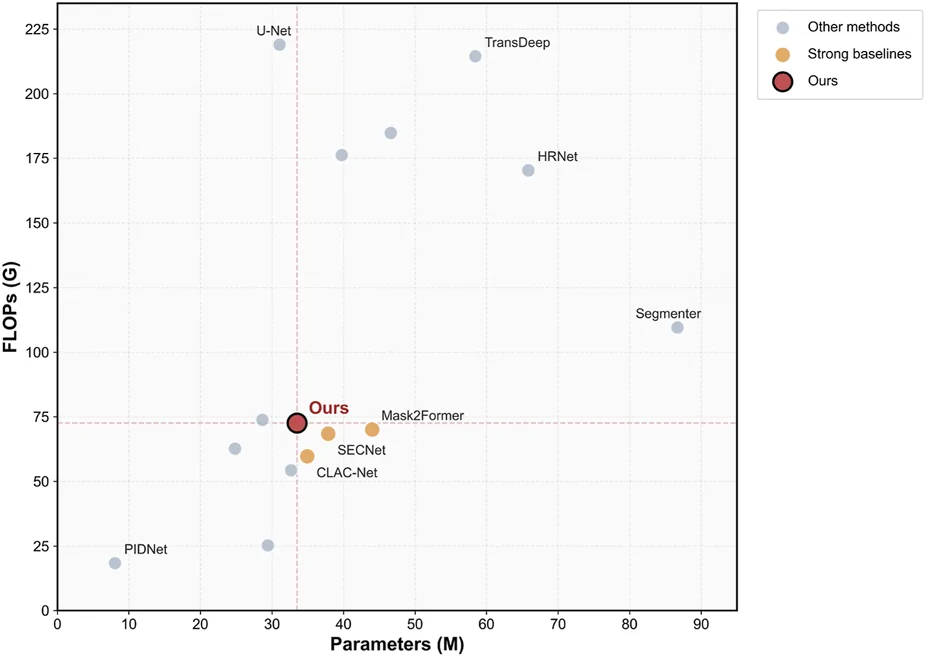
Model complexity comparison in terms of parameter number and floating-point operations (FLOPs) under a fixed 512×512 input setting. The proposed method achieves a moderate computational cost compared with the strongest baselines, while maintaining substantially lower FLOPs than several large convolutional and Transformer-based segmentation models.

As shown in Table 7, both ASPC and CCBR achieve stable performance gains across different evaluation settings, indicating that semantic prototype calibration and class-conditional boundary refinement effectively complement benign–malignant-aware segmentation of thyroid nodules, with the former improving category discrimination and the latter improving boundary correction. On the TN3K dataset, the baseline achieves mIoU and mDice values of 0.5846 and 0.6985, respectively. After introducing ASPC, these values increase to 0.6113 and 0.7221, demonstrating that category prototypes can enhance semantic separability among the background, benign nodules, and malignant nodules. After further introducing CCBR, mIoU and mDice reach 0.6266 and 0.7364, respectively, indicating that boundary-uncertainty modeling and foreground boundary responses can effectively improve the segmentation quality of nodule contour regions. In the supervised case-level, in-house clinical setting, introducing ASPC alone leads to a more pronounced improvement in mPrecision, while introducing CCBR alone achieves more prominent gains in mIoU and mDice. This suggests that ASPC is more effective at reducing benign–malignant category misclassification, whereas CCBR mainly improves regional overlap and structural consistency in weak-boundary regions of clinical images. When the two modules are introduced simultaneously, the model achieves the best results across all metrics in both settings. Specifically, PA, mIoU, mDice, and mPrecision reach 0.9449, 0.6427, 0.7517, and 0.8147 on the TN3K dataset, and 0.8521, 0.6124, 0.7328, and 0.7996 on the supervised case-level, in-house clinical setting, respectively. These results indicate that ASPC and CCBR have a complementary relationship: the former provides stable category-level semantic references, while the latter performs adaptive correction for ambiguous boundaries and high-uncertainty regions. Together, they improve the segmentation accuracy, robustness, and clinical-domain adaptability of the model on both the public dataset and real clinical data. Finally, a bubble chart is provided to illustrate the model’s parameters and floating-point operations per second (FLOPS), as shown in Figure 5.

### 4.5 Qualitative experimental results

**FIGURE 6:**
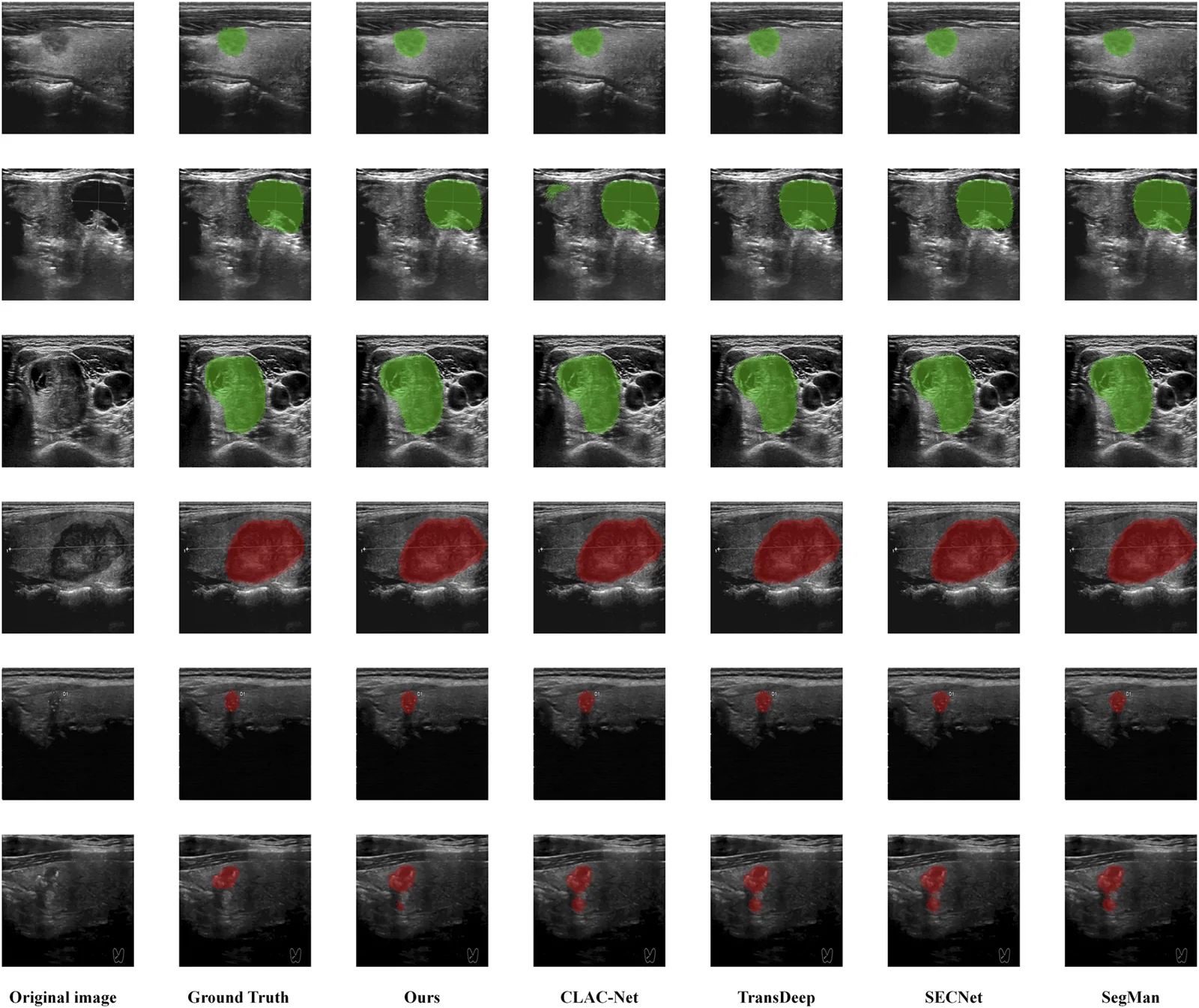
Qualitative comparison of segmentation results obtained by different models on thyroid ultrasound images, where green indicates benign nodule regions, and red indicates malignant nodule regions.

To further intuitively evaluate the practical performance of the proposed method in the benign–malignant-aware segmentation task for thyroid ultrasound images, this study conducts a visual analysis on test set samples and presents the original ultrasound images, expert annotation results, and model prediction results. Unlike quantitative metrics alone, qualitative experiments can more clearly reflect the model’s robustness in nodule boundary localization, weak-contrast region recognition, benign–malignant region discrimination, and complex echo backgrounds. The subsequent visualization results further demonstrate the effectiveness of the proposed method from the perspectives of segmentation contour consistency, lesion-region coverage completeness, and the rationality of model attention regions. First, the qualitative experimental results on the TN3K dataset are presented in Figure 6.

**FIGURE 7:**
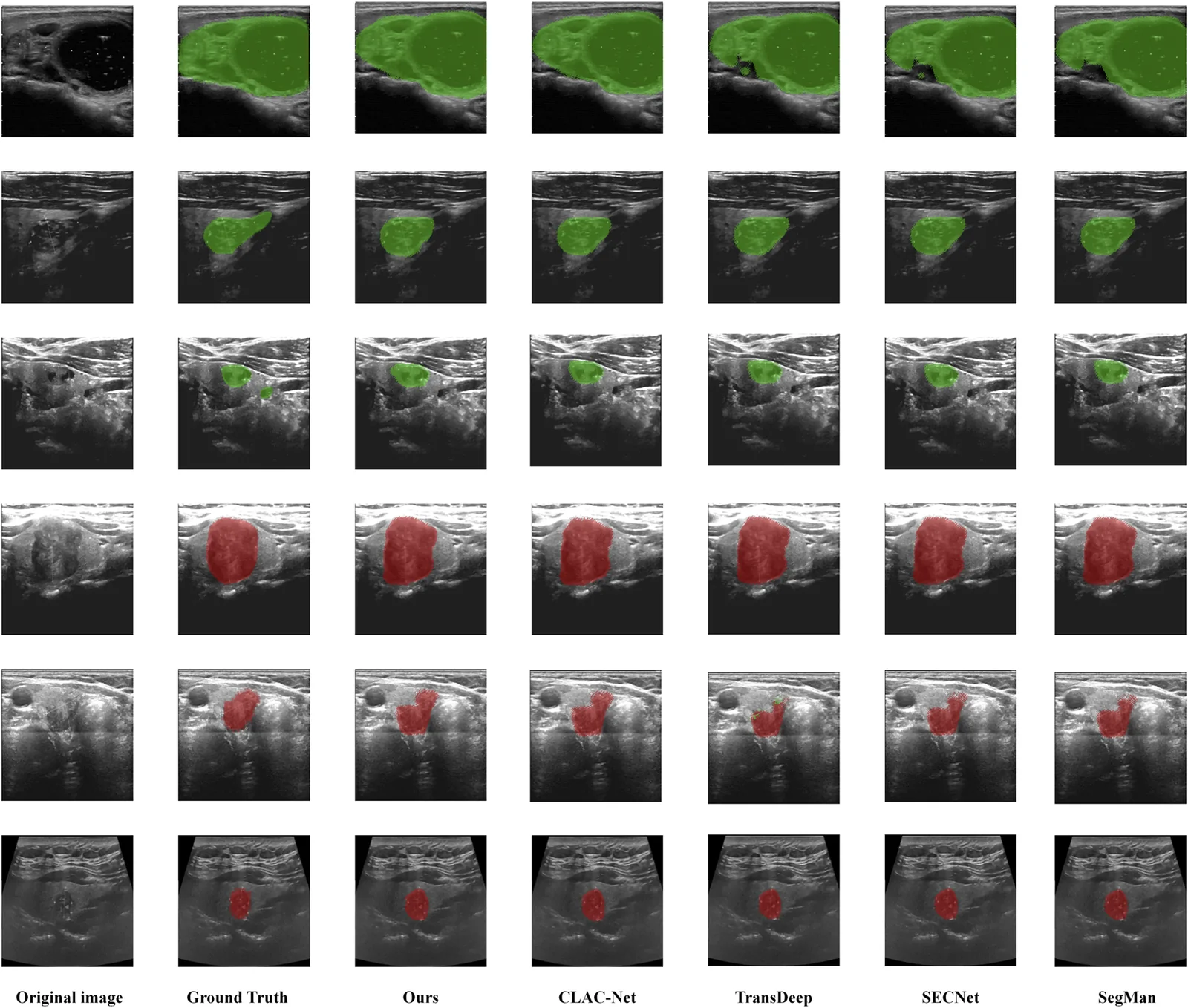
Qualitative comparison of segmentation results obtained by different models on the external clinical validation dataset, where green indicates benign nodule regions, and red indicates malignant nodule regions.

As shown in the figure, the proposed method achieves segmentation results that are closer to expert annotations across thyroid nodule samples with different sizes, morphologies, and echo backgrounds, especially showing better contour preservation ability in cases with ambiguous nodule boundaries, heterogeneous internal textures, and small lesion regions. Compared with CLAC-Net, TransDeep, SECNet, and SegMan, the predicted regions of the proposed method are more consistent with the ground truth in terms of location, shape, and edge continuity. This indicates that the category-level semantic constraint provided by ASPC helps stabilize discrimination between benign and malignant regions, while CCBR refines boundary-uncertain regions to reduce over-segmentation and under-segmentation, thereby improving the visual segmentation quality of the model in complex ultrasound scenarios. This article presents the qualitative experimental results of the clinical validation dataset, as shown in Figure 7.

As shown in the figure, the ultrasound images in the external clinical validation dataset exhibit more obvious imaging differences, ambiguous boundaries, and tissue echo interference. The compared models are prone to boundary deviation, regional omission, or local over-segmentation in some samples. It should be noted that the slightly jagged or blurred appearance of some predicted contours is partly related to the discrete pixel-wise mask representation, image resizing to a fixed resolution, and the inherently low-contrast and fuzzy boundary characteristics of thyroid ultrasound images. Therefore, the visualized contours should be interpreted together with quantitative boundary-oriented metrics rather than judged solely by visual smoothness. In contrast, the proposed method maintains more stable region coverage and contour consistency in benign large nodules, malignant irregular nodules, and small-scale lesions, and its prediction results are closer to expert annotations. This observation is also consistent with the HD95 results reported above, which further indicate that the proposed method reduces boundary localization errors compared with the baseline and other comparison methods. These results indicate that the category prototype constraints provided by ASPC can alleviate semantic drift in clinical data, while the adaptive correction of uncertain boundaries by CCBR further improves the segmentation robustness of the model in real clinical ultrasound scenarios.

### 4.6 Gradient class activation mapping experimental results

**FIGURE 8:**
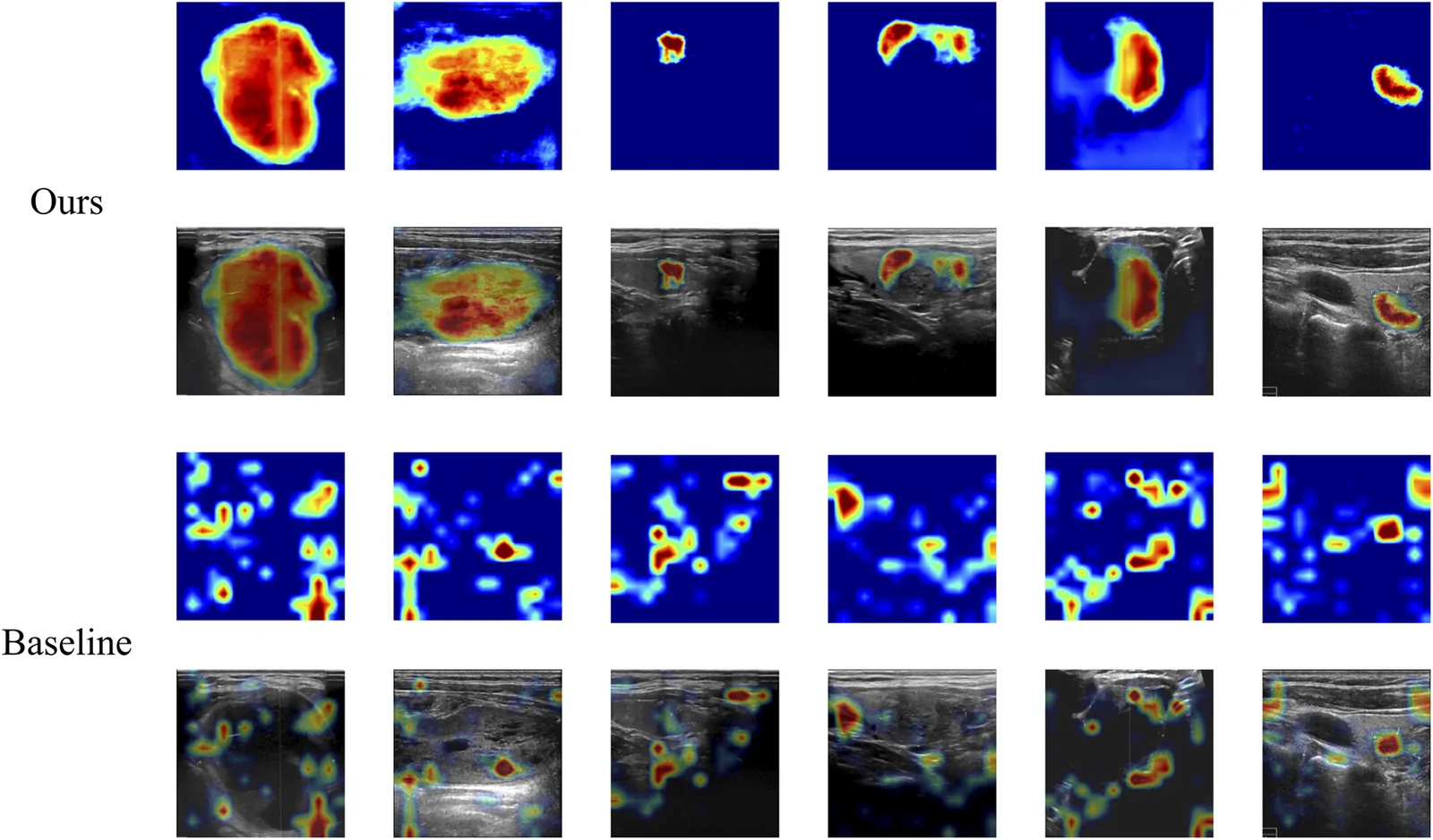
Experimental results of the proposed algorithm and the baseline algorithm in Grad-CAM.

To further analyze the attention regions and the discriminative basis of the model during benign–malignant-aware segmentation of thyroid ultrasound images, this study introduces the gradient class activation mapping (Grad-CAM) method to interpret feature responses from different models. Unlike quantitative metrics and segmentation results, Grad-CAM reflects the main image regions attended to by the model during inference from the perspective of feature activation, thereby helping determine whether the model focuses on the real nodule regions, boundary structures, and diagnostically meaningful local textures. Through this experiment, it is possible to further verify whether the proposed method can form a more reasonable lesion-attention pattern during feature learning, and to provide intuitive evidence for the interpretability of the model in medical image analysis tasks. The experimental results are shown in Figure 8.

As shown in the figure, the Grad-CAM responses of the proposed method are mainly concentrated on the main thyroid nodule regions and their surrounding boundaries. The high-response regions in the heatmaps show relatively good consistency with the lesion locations, indicating that the model tends to pay more attention to diagnostically meaningful nodule morphology, internal echo patterns, and edge structures during benign–malignant-aware segmentation. In contrast, the baseline activation responses are more scattered and tend to appear in background tissues, irrelevant textures, or local noise regions, suggesting that its feature discrimination process may lack stable lesion-region constraints. These results provide supplementary qualitative evidence that the category-level semantic prototypes introduced by ASPC can guide the model to form clearer lesion-related semantic attention, while CCBR further enhances the model’s perception of boundary-uncertain regions. As a result, the final feature responses become more concentrated, continuous, and medically interpretable. It should be noted that Grad-CAM is used here only as a qualitative interpretability visualization. The main effectiveness of the proposed method is primarily supported by the quantitative segmentation metrics, boundary-oriented evaluation, and ablation results.

### 4.7 ASPC prototype dimension sensitivity experiment

**FIGURE 9:**
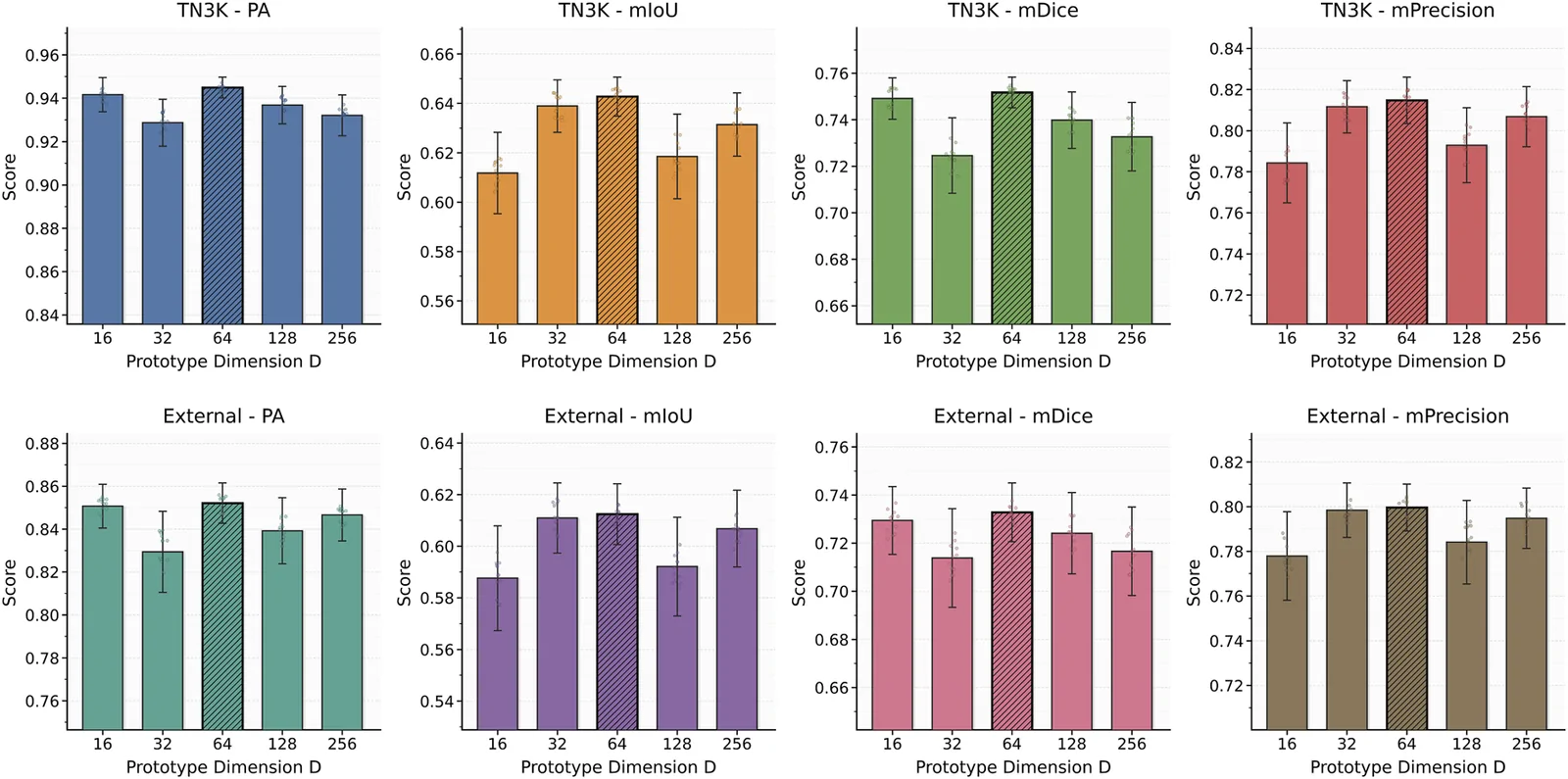
The ASPC prototype dimension sensitivity experiment results demonstrate the performance changes of the model on the TN3K dataset and external clinical validation datasets under different prototype embedding dimensions.

To analyze how prototype dimensions in the ASPC module affect the representation ability of the model, this study further conducts an ASPC prototype dimension sensitivity experiment. By adjusting the dimensional setting of the category prototype embedding space, this experiment investigates the stability of modeling semantic differences among background, benign nodules, and malignant nodules across different prototype capacities, thereby providing a basis for subsequent structural parameter selection. The experimental results are shown in Figure 9.

As shown in the figure, the ASPC prototype dimension clearly influences model performance. However, the changing trends across different metrics and datasets are not simply linear, indicating that a larger prototype dimension does not necessarily lead to better performance. When the prototype dimension is relatively low, the representational capacity of category prototypes is limited, making it difficult to fully characterize the fine-grained semantic differences among the background, benign nodules, and malignant nodules. As a result, the performance of some metrics is constrained. As the prototype dimension continues to increase, redundant features and unstable responses may also be introduced, leading to fluctuations in some metrics, even as the model obtains stronger semantic representation ability. Overall, a medium-dimensional setting achieves a better balance between semantic representation ability and feature compactness, making it more suitable for prototype modeling of benign and malignant nodule regions in thyroid ultrasound images.

Further comparison between the TN3K dataset and the external clinical validation dataset shows that the performance fluctuations on the external clinical data are more obvious. This indicates that imaging differences, ambiguous boundaries, and tissue echo interference in real clinical ultrasound images can amplify the effect of prototype dimension selection on model generalization ability. An appropriate prototype dimension can provide stable semantic references across categories and help the model alleviate category representation drift across different data sources. However, either an excessively small or excessively large prototype space may weaken this calibration effect. This experiment demonstrates that the prototype dimension in ASPC is an important parameter that affects the semantic discrimination ability and clinical generalization stability of the model, and a reasonable setting of this parameter helps improve the overall performance and robustness in benign–malignant-aware segmentation.

### 4.8 Prototype fusion weight sensitivity experiment

**FIGURE 10:**
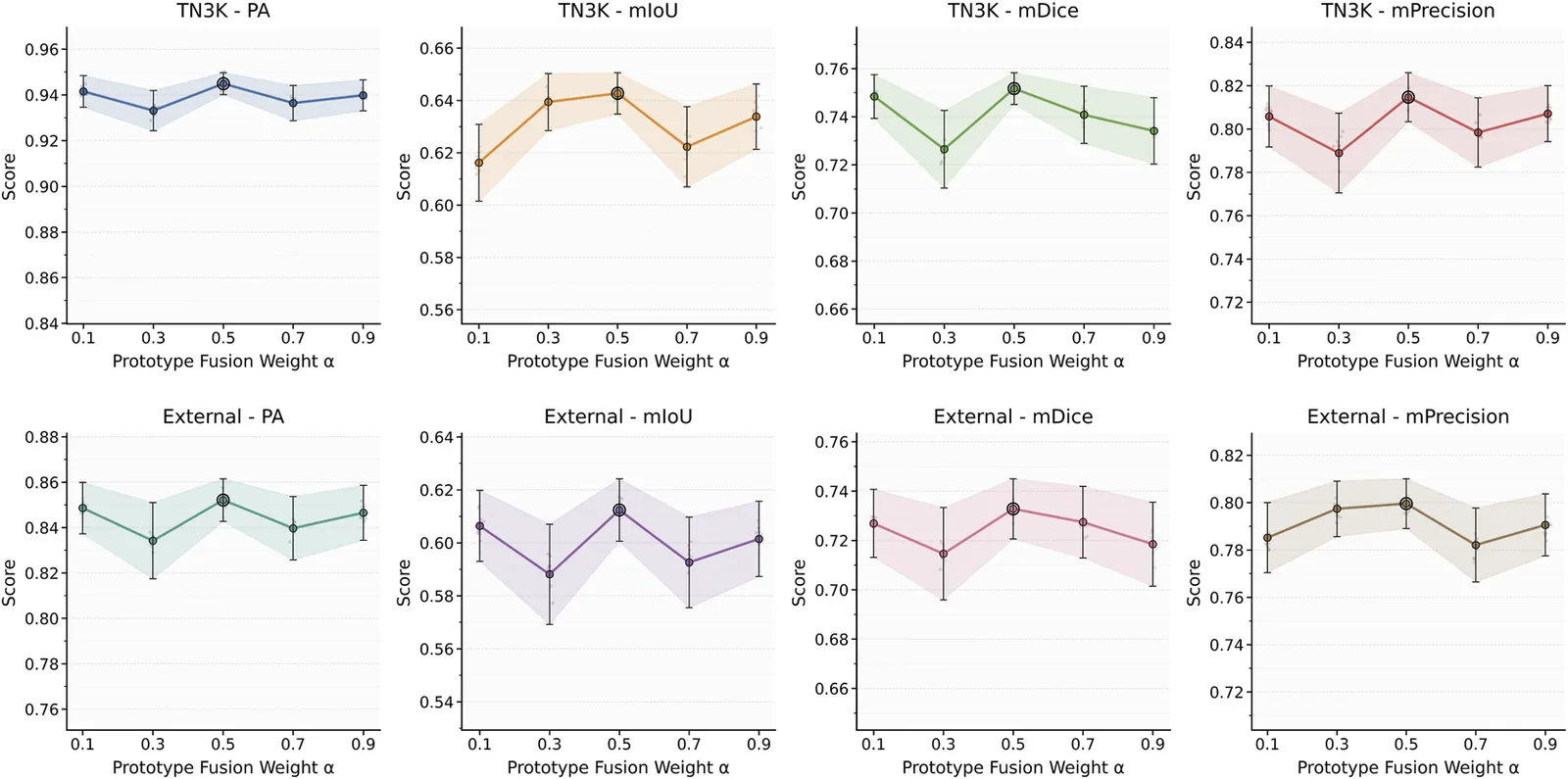
Sensitivity experiment results of the prototype fusion weight α , showing the performance variations of the model on the TN3K dataset and the external clinical validation dataset under different prototype response fusion intensities.

To further analyze the role of prototype responses in overall prediction, this study designs a sensitivity experiment on the prototype fusion weight α and investigates the performance variations of the model under different fusion intensities. By adjusting the contribution ratio of the ASPC prototype-calibrated response in the final segmentation logits, this experiment analyzes the synergistic relationship between semantic prototype constraints and basic predictions. Through this experiment, the effectiveness of the prototype injection strategy in the benign–malignant-aware segmentation of thyroid nodules can be further evaluated, providing a reference for parameter setting. The experimental results are shown in Figure 10.

As shown in the figure, the prototype fusion weight α has a clear influence on model performance, but the trends across different datasets and metrics are not entirely consistent. This indicates that the relationship between prototype responses and basic predictions is not a simple linear enhancement. When α is relatively small, the category prototype information generated by ASPC cannot sufficiently participate in the final prediction, and the semantic calibration effect of the model on benign nodules, malignant nodules, and background regions is relatively limited. When α is excessively large, the prototype response may impose overly strong intervention on the SegFormer basic prediction, thereby affecting local boundary details and the original spatial structure to some extent. Overall, a moderate fusion weight can better balance the basic segmentation logits and the prototype-calibrated logits, enabling the model to simultaneously preserve the spatial localization ability of the backbone network and the category-level semantic constraint ability of ASPC. These results indicate that α is an important parameter that affects the synergistic effect between ASPC and the overall segmentation framework, and a reasonable setting of this weight helps improve the stability and generalization ability of benign–malignant-aware segmentation of thyroid nodules.

### 4.9 External verification experimental results

To further clarify the evaluation protocol, an additional external verification experiment was conducted under three settings. The first setting trained the model on the TN3K training set and evaluated it on the TN3K test set, following the public dataset protocol. The second setting trained the model only on TN3K and directly tested it on the in-house clinical test set without fine-tuning to evaluate cross-source transferability. The third setting trained the model on both the TN3K training set and the in-house training set and then evaluated it on the test set of the external clinical validation dataset under a strict case-level split, thereby assessing the effect of in-house clinical adaptation. All results are reported as mean ± standard deviation over three independent runs, and the 95% confidence intervals were calculated by case-level bootstrap resampling on the corresponding test set. The experimental results are shown in Table 8.

**TABLE 8:** External transfer and mixed-domain adaptation results on the held-out in-house clinical test set under different training settings. Unlike the in-house supervised evaluation in Tables 5, 6, this experiment evaluates cross-domain transfer from TN3K to the in-house clinical test set and mixed-domain training using TN3K together with the in-house clinical training set.

Experimental Setting	PA Mean ± SD 95% CI	MIoU Mean ± SD 95% CI	MDice Mean ± SD 95% CI	MPrecision Mean ± SD 95% CI
TN3K training → TN3K testing	0.9449 ± 0.0048 [0.9362, 0.9528]	0.6427 ± 0.0079 [0.6276, 0.6579]	0.7517 ± 0.0066 [0.7394, 0.7645]	0.8147 ± 0.0113 [0.7935, 0.8358]
TN3K training → in-house testing without fine-tuning	0.7793 ± 0.0308 [0.7164, 0.8287]	0.5016 ± 0.0385 [0.4329, 0.5718]	0.6374 ± 0.0412 [0.5626, 0.7043]	0.7089 ± 0.0367 [0.6415, 0.7742]
TN3K + in-house training set → case-level, in-house testing	0.8365 ± 0.0196 [0.7898, 0.8761]	0.5847 ± 0.0274 [0.5292, 0.6426]	0.7119 ± 0.0248 [0.6617, 0.7595]	0.7758 ± 0.0281 [0.7196, 0.8249]

The results indicate that the model achieved the best performance under the TN3K internal test setting, while its performance decreased when directly transferred to the in-house clinical test set without fine-tuning. This decline suggests that the in-house ultrasound images exhibit domain differences in acquisition equipment, scanning conditions, image quality, nodule morphology, and boundary ambiguity. After incorporating the in-house training set, the model showed clear improvement on the case-level in-house test set, demonstrating that limited clinical data adaptation can help reduce domain discrepancy and improve segmentation stability in local clinical scenarios. However, the confidence intervals in the in-house settings were wider than those in the TN3K setting, indicating greater uncertainty caused by the small number of test cases and the limited number of malignant samples. Therefore, these results support the potential clinical adaptability of the proposed method, while the conclusion regarding external generalization should remain cautious and requires further validation on larger multicenter datasets.

## 5 Discussion

This study focuses on the problem of benign–malignant-aware segmentation in thyroid ultrasound images and constructs a segmentation framework that integrates adaptive semantic prototype calibration and class-conditional boundary refinement. The experimental results show that the proposed method not only achieves favorable segmentation performance on the TN3K dataset but also shows measurable direct external transfer performance on the in-house clinical test set without fine-tuning. It further improved performance under the supervised in-house clinical adaptation setting, indicating that the framework can, to a certain extent, adapt to challenges arising from different ultrasound imaging conditions, variations in nodule morphology, and real clinical noise. The ASPC module establishes category-level semantic prototypes for the background, benign nodules, and malignant nodules, enabling the model to obtain clearer category references at the pixel level during prediction and thereby alleviating semantic confusion between benign and malignant nodule regions. The CCBR module further combines predictive entropy and foreground boundary responses to adaptively correct low-contrast boundaries, weak-texture regions, and uncertain areas, thereby improving boundary continuity and structural consistency while maintaining the overall region localization ability of the model. The Grad-CAM visualization results also show that the proposed method can focus more intensively on the main nodule regions and their boundaries, reducing responses to irrelevant background tissues and local noise, thereby enhancing the medical interpretability of the model outputs. These results demonstrate that, in thyroid ultrasound image analysis, combining category-level semantic constraints with boundary-uncertainty modeling simultaneously improves segmentation accuracy, benign–malignant region discrimination, and clinical-domain adaptability.

Although the proposed method achieves favorable experimental results, it has certain limitations. First, the scales of the TN3K dataset and the clinical validation dataset remain limited, especially given the relatively small number of malignant nodule samples, which may affect the model’s ability to sufficiently learn the morphological diversity of minority classes. In particular, the case-level, in-house test set contained only three malignant cases, and this scarcity of malignant samples may increase statistical uncertainty and requires cautious interpretation of the clinical transfer and adaptation results. Moreover, the reported mean ± standard deviation values are derived from repeated experiments with different random seeds and mainly reflect training randomness rather than case-level confidence intervals; therefore, malignant-class metrics may still have relatively large case-level uncertainty due to the limited number of malignant cases. Second, although the in-house clinical data and direct external transfer setting can preliminarily reflect the generalization ability of the model, the source range and equipment types must be further expanded. Therefore, future validation incorporating more centers, more devices, and larger-scale cases is still required. Third, the proposed method is based on two-dimensional ultrasound images and has not yet fully utilized multimodal data such as video sequences, elastography, color Doppler imaging, or clinicopathological information. As a result, there remains room for improvement in the comprehensive assessment of some complex cases.

In practical applications, the proposed method can serve as a lesion localization and benign–malignant region indication tool in thyroid ultrasound-assisted diagnosis systems, providing doctors with auxiliary information such as nodule contours, suspicious regions, and model attention areas, thereby improving image interpretation efficiency and segmentation consistency. However, the method should currently be positioned as an auxiliary decision-making tool rather than a replacement for the final diagnosis made by doctors. Future studies can further introduce large-scale multicenter data, cross-device domain adaptation strategies, and multimodal clinical information fusion mechanisms, while combining model uncertainty estimation and human–machine interactive correction to improve the safety, reliability, and application value of the model in real clinical workflows.

In addition, this study has not yet been fully aligned with diagnostic reporting frameworks such as CLAIM or STARD-AI, because the current work is primarily positioned as a benign–malignant-aware segmentation study rather than a complete diagnostic prediction system. Although calibration curves, decision-curve analysis, and PR-AUC are valuable for evaluating diagnostic reliability and clinical utility, these analyses may be statistically unstable on the in-house clinical test set because it contains only three malignant cases. Therefore, the present study does not use these diagnostic-level analyses as the main evidence for model effectiveness. Future studies should further conduct calibration analysis, decision-curve analysis, PR-AUC evaluation, and reporting-framework-based validation on larger, adequately powered, and multicenter clinical datasets.

## 6 Conclusion

This study addresses the challenges of ambiguous nodule boundaries, subtle differences between benign and malignant regions, and limited clinical-domain transfer and adaptation in thyroid ultrasound images. To this end, a benign–malignant-aware segmentation framework integrating adaptive semantic prototype calibration and class-conditional boundary refinement is proposed. The proposed method adopts SegFormer as the basic backbone network. Through the ASPC module, explicit category prototypes are established for the background, benign nodules, and malignant nodules, thereby enhancing pixel-level semantic discrimination. Meanwhile, the CCBR module integrates predictive uncertainty and foreground boundary responses to adaptively correct nodule edges and category-confusing regions. Experimental results demonstrate that the proposed method achieves favorable segmentation performance on the TN3K dataset, demonstrates direct external transfer performance on the in-house clinical test set without fine-tuning, and further improves performance under the supervised in-house clinical adaptation setting. It also shows more stable lesion localization ability and more reasonable feature attention regions in qualitative visualizations and Grad-CAM results. Overall, the proposed method can improve thyroid nodule segmentation accuracy while enhancing benign–malignant region discrimination and model interpretability, providing a technically promising solution for intelligent assisted diagnosis in thyroid ultrasound.

## Data Availability

The original contributions presented in the study are included in the article/Supplementary Material; further inquiries can be directed to the corresponding author.
