# Supplementary material for: Intelligent segmentation of thyroid nodule ultrasound images based on benign–malignant-aware semantic prototype calibration and class-conditional boundary refinement
Source: Front Cell Dev Biol. 2026 Aug 21;14:1879094. doi: 10.3389/fcell.2026.1879094 (PMC13541914; doi:10.3389/fcell.2026.1879094)
Supplement: Supplementary file 1 [file Table1.docx]

Binary and three-class foreground localization control experiments on the TN3K dataset.

| **Method** | **Task Setting** | **PA** | **mIoU** | **mDice** | **mPrecision** | **FG Dice** | **FG IoU** |
| --- | --- | --- | --- | --- | --- | --- | --- |
| SegFormer-B0 Baseline | Binary nodule segmentation | 0.9296 ± 0.0072 | 0.8164 ± 0.0115 | 0.8872 ± 0.0096 | 0.8468 ± 0.0143 | 0.8427 ± 0.0128 | 0.7281 ± 0.0186 |
| SegFormer-B0 Baseline | Three-class class-conditional segmentation | 0.9128 ± 0.0069 | 0.5846 ± 0.0107 | 0.6985 ± 0.0083 | 0.7589 ± 0.0146 | 0.8216 ± 0.0149 | 0.6972 ± 0.0205 |
| Ours | Binary nodule segmentation | **0.9538 ± 0.0049** | **0.8387 ± 0.0086** | **0.9041 ± 0.0068** | **0.8725 ± 0.0112** | **0.8619 ± 0.0097** | **0.7569 ± 0.0148** |
| Ours | Three-class class-conditional segmentation | 0.9449 ± 0.0048 | 0.6427 ± 0.0079 | 0.7517 ± 0.0066 | 0.8147 ± 0.0113 | 0.8449 ± 0.0093 | 0.7314 ± 0.0117 |

Appendix boundary-oriented ablation results with different boundary refinement or supervision strategies.

| **Dataset** | **Method** | **Boundary Strategy** | **HD95↓** |
| --- | --- | --- | --- |
| **TN3K** | Baseline | None | 39.84 ± 3.27 |
|  | Baseline + Contour loss | Explicit contour supervision | 33.79 ± 2.96 |
|  | Baseline + Hausdorff loss | Explicit distance-based supervision | 32.54 ± 2.73 |
|  | Baseline + Level-set loss | Explicit shape-level supervision | 34.18 ± 3.14 |
|  | Baseline + SDM loss | Explicit signed-distance supervision | **30.48 ± 2.29** |
|  | Baseline + CCBR | Self-generated uncertainty-boundary refinement | 31.16 ± 2.43 |
| **Supervised in-house clinical setting** | Baseline | None | 33.68 ± 3.74 |
|  | Baseline + Contour loss | Explicit contour supervision | 31.27 ± 3.42 |
|  | Baseline + Hausdorff loss | Explicit distance-based supervision | 28.86 ± 3.19 |
|  | Baseline + Level-set loss | Explicit shape-level supervision | 30.14 ± 3.58 |
|  | Baseline + SDM loss | Explicit signed-distance supervision | 27.92 ± 2.84 |
|  | Baseline + CCBR | Self-generated uncertainty-boundary refinement | **27.71 ± 2.95** |

Paired statistical comparison between the proposed method and the strongest baseline models. For the TN3K dataset, paired bootstrap resampling and permutation testing were performed at the image level. For the in-house supervised setting, all resampling and paired tests were performed at the case level to avoid treating multiple images from the same case as independent samples. The reported effect size denotes the paired performance difference between Ours and each baseline. For PA, mIoU, mDice, mPrecision, FG Dice, FG IoU, Benign Dice, Malignant Dice, and malignancy sensitivity, the effect size is defined as Ours minus baseline; for HD95, the effect size is defined as baseline minus Ours, so that a positive value consistently indicates better performance of the proposed method. Values in parentheses denote the 95% confidence interval estimated by paired bootstrap resampling, and the corresponding p values were obtained from paired permutation tests.

| **Dataset** | **Metric** | **Ours vs. CLAC-Net** | **Ours vs. SECNet** | **Ours vs. Mask2Former** |
| --- | --- | --- | --- | --- |
| TN3K | PA | 0.0164 (0.0087, 0.0242); p<0.001 | 0.0204 (0.0117, 0.0291); p<0.001 | 0.0239 (0.0182, 0.0295); p<0.001 |
| TN3K | mIoU | 0.0198 (0.0103, 0.0294); p<0.001 | 0.0344 (0.0271, 0.0416); p<0.001 | 0.0365 (0.0293, 0.0437); p<0.001 |
| TN3K | mDice | 0.0179 (0.0084, 0.0275); p<0.001 | 0.0249 (0.0190, 0.0309); p<0.001 | 0.0409 (0.0341, 0.0477); p<0.001 |
| TN3K | mPrecision | 0.0179 (0.0116, 0.0241); p<0.001 | 0.0230 (0.0168, 0.0292); p<0.001 | 0.0335 (0.0239, 0.0431); p<0.001 |
| TN3K | HD95 | 5.70 (3.28, 8.12); p<0.001 | 8.12 (5.66, 10.59); p<0.001 | 9.17 (7.09, 11.24); p<0.001 |
| TN3K | FG Dice | 0.0219 (0.0133, 0.0304); p<0.001 | 0.0325 (0.0229, 0.0421); p<0.001 | 0.0418 (0.0330, 0.0507); p<0.001 |
| TN3K | FG IoU | 0.0224 (0.0169, 0.0278); p<0.001 | 0.0502 (0.0390, 0.0614); p<0.001 | 0.0659 (0.0552, 0.0767); p<0.001 |
| TN3K | Benign Dice | 0.0197 (0.0135, 0.0259); p<0.001 | 0.0357 (0.0283, 0.0431); p<0.001 | 0.0463 (0.0372, 0.0555); p<0.001 |
| TN3K | Malignant Dice | 0.0257 (0.0153, 0.0362); p<0.001 | 0.0414 (0.0311, 0.0516); p<0.001 | 0.0479 (0.0368, 0.0590); p<0.001 |
| TN3K | Malig. Sens. | 0.0344 (0.0263, 0.0425); p<0.001 | 0.0501 (0.0393, 0.0608); p<0.001 | 0.0671 (0.0569, 0.0773); p<0.001 |
| In-house supervised | PA | 0.0201 (0.0042, 0.0360); p=0.016 | 0.0180 (0.0059, 0.0300); p=0.003 | 0.0199 (0.0001, 0.0398); p=0.045 |
| In-house supervised | mIoU | 0.0194 (0.0042, 0.0346); p=0.015 | 0.0243 (0.0037, 0.0449); p=0.021 | 0.0169 (0.0008, 0.0331); p=0.047 |
| In-house supervised | mDice | 0.0167 (-0.0002, 0.0336); p=0.065 | 0.0186 (0.0011, 0.0361); p=0.048 | 0.0173 (0.0037, 0.0309); p=0.012 |
| In-house supervised | mPrecision | 0.0152 (0.0020, 0.0285); p=0.040 | 0.0204 (0.0052, 0.0355); p=0.010 | 0.0165 (0.0014, 0.0317); p=0.033 |
| In-house supervised | HD95 | 4.76 (1.88, 7.63); p=0.001 | 6.53 (4.03, 9.04); p<0.001 | 5.68 (2.13, 9.24); p=0.002 |
| In-house supervised | FG Dice | 0.0164 (0.0013, 0.0316); p=0.035 | 0.0241 (0.0124, 0.0359); p<0.001 | 0.0177 (0.0024, 0.0330); p=0.032 |
| In-house supervised | FG IoU | 0.0181 (-0.0013, 0.0374); p=0.058 | 0.0309 (0.0112, 0.0506); p=0.002 | 0.0266 (0.0122, 0.0409); p<0.001 |
| In-house supervised | Benign Dice | 0.0239 (0.0061, 0.0417); p=0.007 | 0.0349 (0.0136, 0.0561); p=0.001 | 0.0207 (0.0034, 0.0379); p=0.020 |
| In-house supervised | Malignant Dice | 0.0178 (0.0017, 0.0339); p=0.041 | 0.0253 (0.0088, 0.0417); p=0.003 | 0.0112 (-0.0037, 0.0260); p=0.177 |
| In-house supervised | Malig. Sens. | 0.0276 (0.0039, 0.0512); p=0.028 | 0.0417 (0.0200, 0.0634); p<0.001 | 0.0417 (0.0193, 0.0640); p<0.001 |
